# Quinoline-Based Molecules Targeting c-Met, EGF, and VEGF Receptors and the Proteins Involved in Related Carcinogenic Pathways

**DOI:** 10.3390/molecules25184279

**Published:** 2020-09-18

**Authors:** Annamaria Martorana, Gabriele La Monica, Antonino Lauria

**Affiliations:** Dipartimento di Scienze e Tecnologie Biologiche Chimiche e Farmaceutiche “STEBICEF”–University of Palermo, Viale delle Scienze—Ed. 17, 90128 Palermo, Italy; annamaria.martorana@unipa.it (A.M.); gabriele.lamonica01@unipa.it (G.L.M.)

**Keywords:** quinoline, antiproliferative compounds, targeted therapy, SAR studies, carcinogenic pathways, kinases modulators, biological data

## Abstract

The quinoline ring system has long been known as a versatile nucleus in the design and synthesis of biologically active compounds. Currently, more than one hundred quinoline compounds have been approved in therapy as antimicrobial, local anaesthetic, antipsychotic, and anticancer drugs. In drug discovery, indeed, over the last few years, an increase in the publication of papers and patents about quinoline derivatives possessing antiproliferative properties has been observed. This trend can be justified by the versatility and accessibility of the quinoline scaffold, from which new derivatives can be easily designed and synthesized. Within the numerous quinoline small molecules developed as antiproliferative drugs, this review is focused on compounds effective on c-Met, VEGF (vascular endothelial growth factor), and EGF (epidermal growth factor) receptors, pivotal targets for the activation of important carcinogenic pathways (Ras/Raf/MEK and PI3K/AkT/mTOR). These signalling cascades are closely connected and regulate the survival processes in the cell, such as proliferation, apoptosis, differentiation, and angiogenesis. The antiproliferative biological data of remarkable quinoline compounds have been analysed, confirming the pivotal importance of this ring system in the efficacy of several approved drugs. Furthermore, in view of an SAR (structure-activity relationship) study, the most recurrent ligand–protein interactions of the reviewed molecules are summarized.

## 1. Introduction

**Figure 1:**
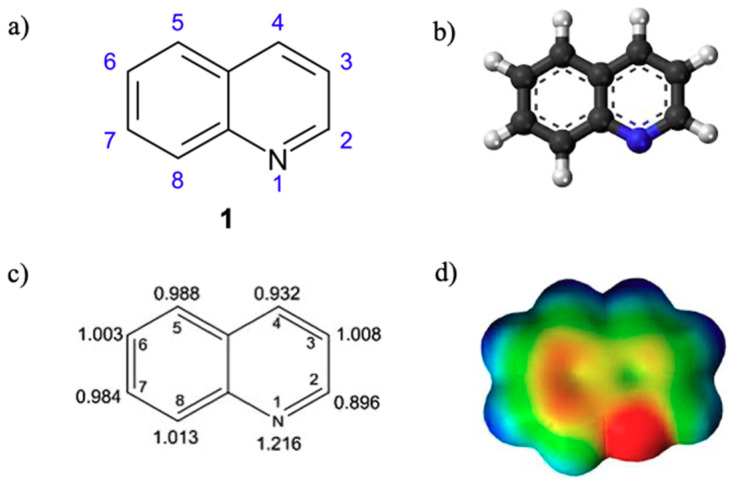
(**a**) Quinoline **1** or 1-aza-naphthalene and benzo[*b*]pyridine; (**b**) quinoline 3D structure; (**c**) quinoline electron density; (**d**) electron density encoded with the electrostatic potential.

Quinoline **1** is an important and versatile nucleus recurrent in several natural and pharmacologically active molecules. Due to the presence of nitrogen, which withdraws electrons by resonance, this fused aromatic scaffold is an electron-deficient ring system (Figure 1), displaying weak tertiary base properties. Quinoline reacts with similar behaviour of pyridine and allows both nucleophilic and electrophilic substitution reactions. From a biological point of view, the nontoxic effect to humans, via inhalation and oral absorption, makes the 1-aza-naphthalene an interesting scaffold to be studied for the development of more selective drugs [1].

Quinoline and its related derivatives exhibit a broad spectrum of pharmacological activities from antibacterial, antifungal, antimalarial, anthelmintic, local anaesthetic, antipsychotic, and anticancer [2]. Especially in the research of new anticancer agents, quinoline is one of the most important scaffolds in drug discovery. Indeed, over the last few years, an increase in the publication of papers about quinoline derivatives possessing antiproliferative properties has been observed. This trend can be justified by the “druggability”, versatility, and accessibility of the quinoline nucleus [3].

Furthermore, different quinoline small molecules, acting as protein kinases inhibitors, have been approved by the Food and Drug Administration (FDA) for clinical uses [4,5]. In this contest, bosutinib, a potent inhibitor of Abl, was approved in 2012 for the treatment of Philadelphia chromosome-positive chronic myelogenous leukaemia (CML) [6]; lenvatinib, an inhibitor of RET and VEGFR has been employed, since 2015, for the treatment of differentiated thyroid cancers [7]; and neratinib and cabozantinib were authorized for the clinical treatment respectively in 2017 and 2012 [8,9].

Due to the importance of quinoline nucleus in the medicinal chemistry field, the main purpose of this review is to provide an overview of the most relevant anticancer quinoline molecules described in the literature, in the last decade. In particular, the attention has been focused on quinoline drugs and experimental active derivatives, able to interfere with c-Met (mesenchymal-epithelial transition factor), VEGF, and EGF receptors and the correlated proteins of the intracellular signalling pathways. The interest on these targets rises from their crucial involvement in several carcinogenic degenerations. In fact, closely connected by each other, they regulate the survival mechanisms in the cell, such as proliferation, apoptosis, differentiation, and angiogenesis.

In this light, the evaluation of the antiproliferative data and the analysis of most frequent ligand–protein interactions of quinoline-based compounds could support, in a drug discovery process, the search for new and more efficacious multi-targets drugs.

### EGFR, c-MET, and VEGFR Signal Pathways in Cancer Cell

Aberrant receptor tyrosine kinases (RTKs) are deeply involved in cancer progression, and for this reason represent the key oncology therapeutic targets. Among the most studied, in this review the attention has been focused on three crucial growth factor receptors: c-Met, EGFR, and VEGFR [10].

C-Met is a tyrosine kinase receptor belonging to the MET (MNNG HOS transforming gene) family, and is normally expressed on the surfaces of liver, pancreas, prostate, kidney, muscle, and bone marrow cells, where mediates tissue regeneration and wound healing [11,12]. The endogenous ligand of c-Met is HGF/SF (ligand hepatocyte growth factor/scatter factor), that induces signal transduction from the extracellular to the intracellular domain [13]. The kinase catalytic activity triggers the transphosphorylation of the tyrosines Tyr^1234^ and Tyr^1235^, turning on a whole signalling cascade of biological pathways correlated to the cellular growth and replication [14,15]. 

**Figure 2:**
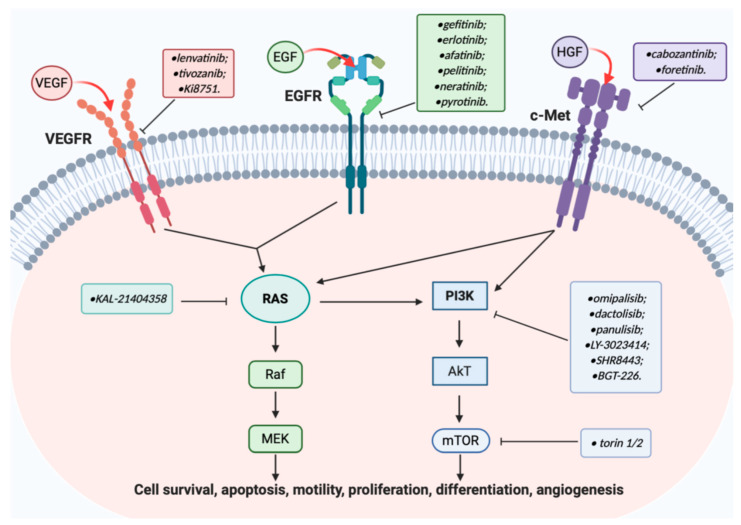
Cross-talk between EGFR, VEGFR, and c-Met signalling pathways; in boxes are reported some quinoline based inhibitors developed up to date.

C-Met is strictly connected with the phosphoinositide 3-kinase (PI3K) a cytosolic family of kinases responsively to phosphorylate the 3’-hydroxyl group of phosphoinositides [16], (Figure 2). After activation by growth factors and/or cytokines, PI3K is able to turn on downstream proteins, such as AkT, a serine-threonine kinase, that consequently controls stimulation of mTOR complex 1 (mTORC1). AkT is responsible for the handling of the cell cycle, through genes transcription activation. Furthermore, another component of these pathways is mTOR complex 2 (mTORC2), that determinates activation of AkT by its phosphorylation. It has been studied that this signalling pathway is upregulated in many cancers and apparently drives the resistance to the typical cytotoxic anticancer drugs. For these reasons PI3K/AkT/mTOR appear attractive targets in cancer targeted therapy [17,18,19].

C-Met intracellular signalling cascade is also correlated to the carcinogenic pathways stimulated by EGFR and VEGFR (Figure 2). The equilibrium in the activation of these three growth factor receptors is at the basis of normal cell survival processes, such as proliferation, apoptosis, differentiation, and angiogenesis [11,20].

EGFR belong to a family of tyrosine kinases receptors and include four members: EGFR (ErbB1), ErbB2, ErbB3, and ErbB4. These receptors are widely distributed in mammalian epithelial cell membranes and normally modulate different processes such as cell proliferation, death, and differentiation. It has been detected that an anomalous over-expression of these proteins has an important role in the formation and development of many types of solid tumours, principally Non-Small Cell Lung (NSCLC), breast, colorectal, head, and neck cancers types [21,22,23].

VEGFR can be secreted by tumour cells and, through its interaction with one of the three tyrosine kinase receptors (VEGFR1-3), it is able to promotes vascular growth and permeability, with an antiapoptotic effect on newly formed cells [24,25]. 

EGFR and/or VEGFR recruits Rat sarcoma (RAS) protein family, with consequent activation of Raf/MEK/ERK and of PI3K/AkT/mTOR signalling cascade, directly activated also by c-Met [16,20]. 

With respect to the aforementioned intracellular effectors, Ras, a membrane-associated guanine nucleotide-binding protein, plays a central role in regulating the cytoplasmatic transduction cascades, especially those of Raf/MEK and PI3K, leading to cell growth (Figure 2) [26,27,28].

## 2. Quinolines as Inhibitors of Carcinogenic Pathways

### 2.1. Quinoline Based Inhibitors of c-Met Receptor

**Figure 3:**
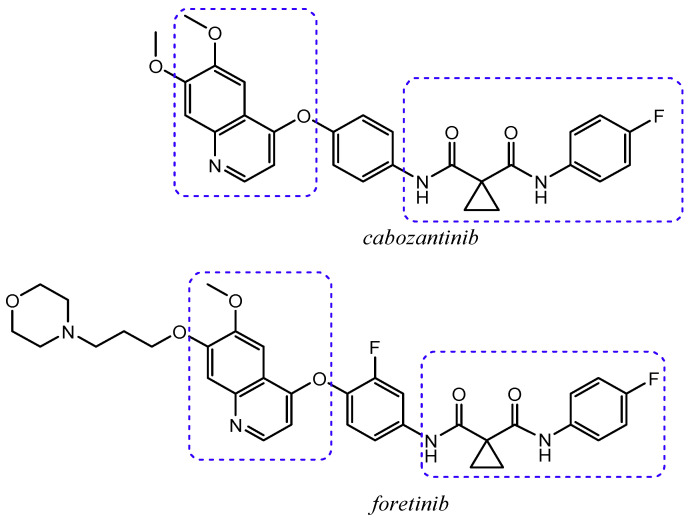
C-Met quinoline inhibitors: *cabozantinib* and *foretinib* (in boxes structure analogies are highlighted).

An alteration of the HGF/c-Met signalling has been detected in liver, pancreatic, breast, ovarian, gastric, and prostate cancers and it contributes to the development, progression, dissociation of cells from its primary site, and distant colonization. Therefore, c-Met is considered an attractive target for cancer therapy [11,12]. Currently, a lot of small molecules with a quinoline nucleus, classified as c-Met inhibitors, have been approved for therapeutic treatments. Among these, we could mention cabozantinib, a multikinase inhibitor administered in the treatment of advanced medullary thyroid cancers [8,29], and foretinib, a drug candidate under experimental evaluation (Figure 3) [3].

**Figure 4:**
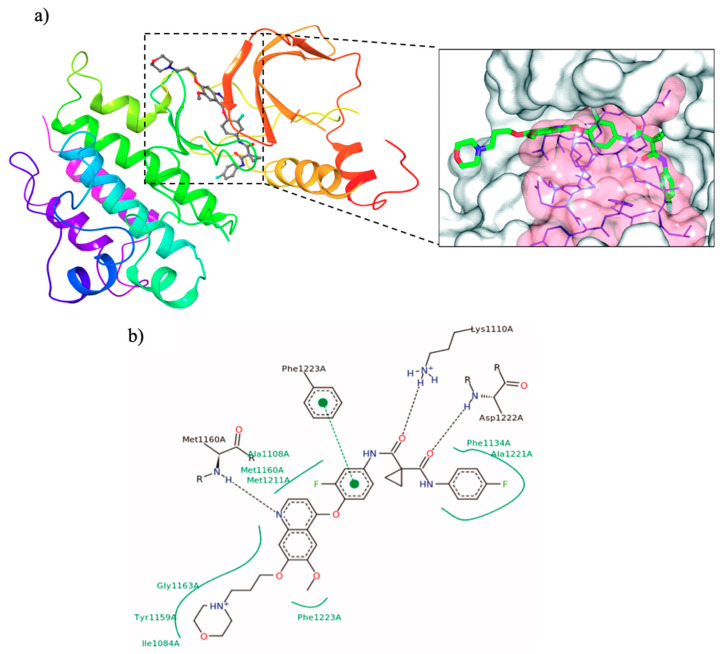
(**a**) crystal structure of kinase domain of c-Met in complex with *foretinib* (PDB ID: 3LQ8). In the box the binding cavity of the receptor occupied by the quinoline small molecule is illustrated (the activation loop is represented with transparent surface) [31]; (**b**) interactions of *foretinib* with amino acid residues of the c-Met active site [32].

From a molecular point of view, all the quinoline-based inhibitors of c-Met interact with the kinase domain of the receptor in the cytosolic side, involving amino acids residues from 1078 to 1345 [13,30]. As an example, in Figure 4a, the crystal structure of c-Met kinase domain in complex with foretinib (PDB id: 3LQ8) is reported: the ligand binds to c-Met in an extended conformation occupying the ATP-binding site and the adjacent deep hydrophobic pocket. The cyclopropane-1,1-dicarboxiamide framework interferes with the Phe^1223^ of the DFG motif, determining the disruption of the catalytic conformation (from DFG-in to DFG out) and the reorganization of the activation loop, which almost entirely envelops the ligand (Figure 4a). In the cavity the reoriented Phe^1223^ forms a π-π stacking with the central fluorophenyl ring, the quinoline nitrogen links with a hydrogen bond the backbone amide of Met^1160^ and also the dicarboxamide moiety forms additional hydrogen bonds with Lys^1110^ and Asp^1222^ (Figure 4b) [30,31,32]. 

**Figure 5:**
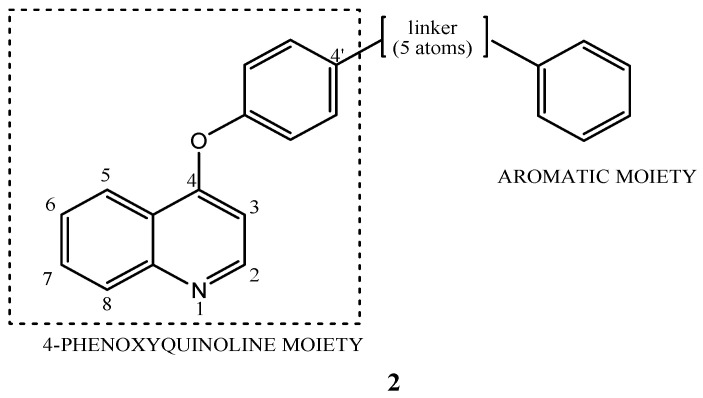
General structure for quinoline c-Met inhibitors as analogues of *cabozantinib* and *foretinib*.

Considering cabozantinib and foretinib as lead compounds, for the design of new quinoline derivatives selectively acting on c-Met, SAR studies highlight the 4-phenoxyquinoline and an aromatic moiety, connected each other by a linker, as the fundamental pharmacophoric portions in the modulation of c-Met tyrosine kinase activity (Figure 5). Generally, the linker should have two important features: (1) the distance of five atoms (the so-called “five atoms regulation”) between the 4-phenoxyquinoline moiety and the aromatic one; (2) the presence of donor and/or acceptor hydrogen-bond groups and at least one amide. These general features are also shown in the structure of cabozantinib and foretinib, in which there is a cyclopropane-1,1-dicarboxiamide framework as linker [33]. 

In the last few years, a lot of more active and selective quinoline derivatives have been synthesized and biologically evaluated by modifying lead compounds (Table 1). The most representative modifications are: (1) at position C-7 of quinoline ring system, generally with the introduction of appropriate substituents to improve solubility, (2) at the linker portion [34], that has been modified by different cyclic/acyclic 5-atoms-analogues with similar electronic features: pyridazinone-3-carboxyamide **3** [35], 3-oxo-3,4-dihydroquinoxaline **4** [36], 1H-imidazole-4-carboxamide or (E)-3-hydrosulfonylacrylamide **5**,**6** [37], 1,2,3-triazole-4-carboxamide **7**,**8** [38,39], 2-imidazolone-4-carboxamide **9** [40], acylhydrazone moiety **10** [41], pyridine/pyrimidine-2-carboxyamide **11** [42], 2-phenylquinoline-4-carboxamide **12** [33], 4-oxo-1,4-dihydrocinnoline-3-carboxamide moiety **13** [43], acyclic semicarbazones **14** [44,45], acylthiourea moiety [46], l,2,4-triazine-3,5-dione **15** [47], 1,8-naphthyridin-2-one **16** [48], 2-oxo-1,2-dihydropiridine-3-carboxamide **17** [49], 4-oxo-1,4-dihydroquinoline-3-carboxamide **18** [50], 5-(aminomethylene)pyrimidine-2,4,6-trione moiety **19** [51], 4-oxo-3,4-dihydrophthalazine-1-carboxamide **20** [52], pyrazolone **21** [53], and (thio)semicarbazone **22** [54]. 

In this regard, after treatment with interesting quinoline compounds, in Table 1 are reported the IC_50_ values (concentration of the compound producing 50% of cell growth inhibition) evaluated against the more responsive human cancer cell lines and the IC_50_ values (concentration of the compound producing 50% of inhibition activity) against the c-Met receptor, (bold data show the values of target compounds lower than that of the positive control in the corresponding experiment).

**Figure 6:**
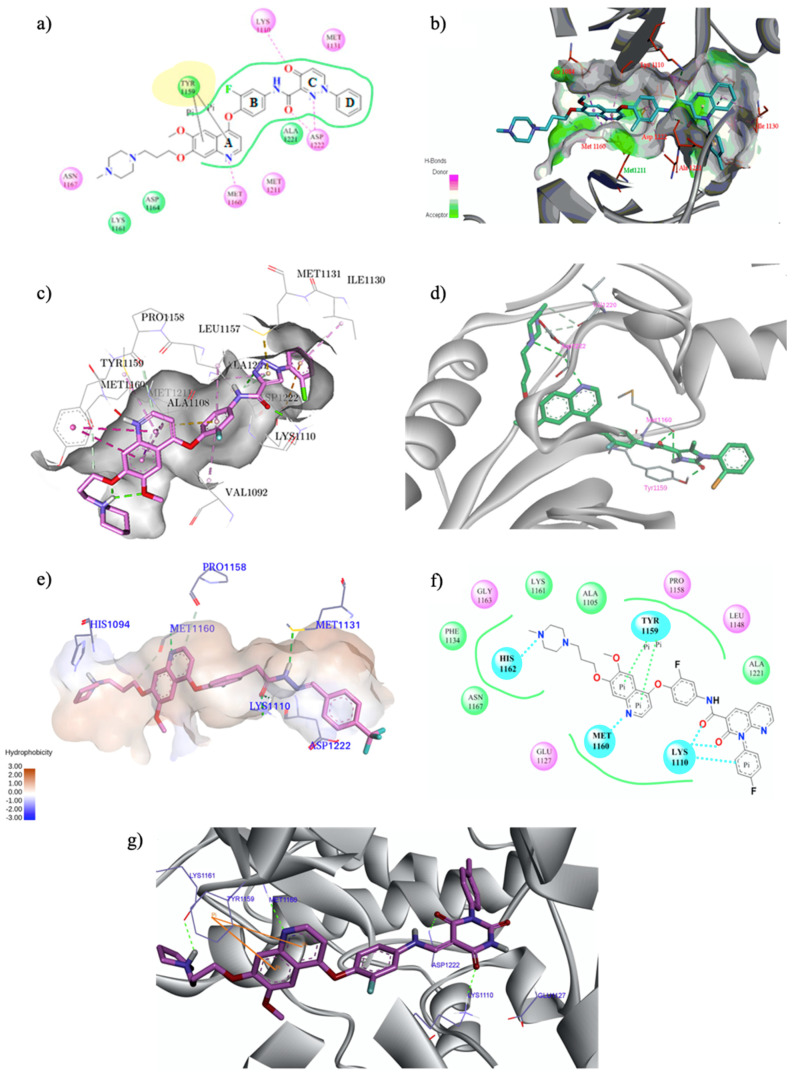
Binding models with c-Met kinase domain of quinoline- based compounds (**a**) **3** [35]; (**b**) **4** [36]; (**c**) **7** [38]; (**d**) **9** [40]; (**e**) **10** [41]; (**f**) **16** [48]; (**g**) **19** [51].

In order to extrapolate important general information about the interactions between ligands and amino acid residues in the active site, the binding mode of the previous cabozantinib/foretinib-like derivatives has been explored through molecular docking simulations, (Figure 6). These studies pointed out π-π stacking interactions between the quinoline ring and Tyr^1159^ and the formation of a hydrogen bond between the lone pair of the quinoline nitrogen and Met^1160^, playing a pivotal role in the stabilization of the ligand-protein binding. Other important interactions occur through hydrogen bonds between the O and N atoms of the different linker regions and amino acids residues as Asp^1222^ and Lys^1110^ [35,36,38,40,41,48,51]. 

**Figure 7:**
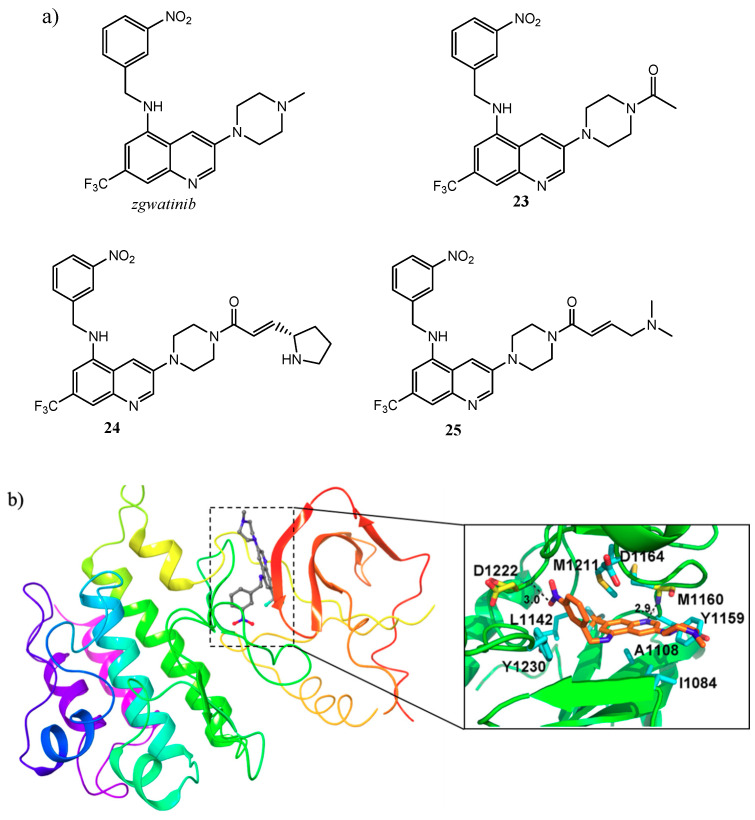
(**a**) structures of a new cluster of 3,5,7-trisubstituted quinoline compounds with promising anticancer activity on c-Met [55,57]; (**b**) crystal structure of *zgwatinib* in complex with the kinase domain of c-Met (residues coloured in cyan form hydrophobic interactions with the ligand, whereas those coloured in yellow formed H-bonds, indicated with black dash lines; PDB id: 4GG5), [56].

In 2011, Wang and co-workers designed a relatively unexplored quinoline chemotype, identifying the lead compound 3,5,7-trisubstituted derivative (zgwatinib) and the quinoline **23** as novel anticancer agents with a potent and selective inhibition activity on c-Met (IC_50_ of 0.93 and 0.95 nM, respectively) (Figure 7a). In particular, compound **23** showed significant antiproliferative effects (IC_50_ in the range 1–5 μM) especially on cancer cell lines characterized by c-Met overexpression (ICMKN45, SNU-5 and H1993), exhibiting a promising PK profile and a remarkable in vivo tumour growth inhibition in human glioblastoma xenograft models [55]. The crystal structure of zgwatinib in complex with the kinase domain of c-Met was determined in a further study, to elucidate the binding mode of this class of compounds. In detail, quinoline nitrogen forms an H-bond with Met^1160^ in the hinge region of the kinase, the trifluoromethyl and the benzylamino groups occupy the hydrophobic pocket, and the nitro substituent forms H-bond with Asp^1222^ (Figure 7b) [56].

Nevertheless, further toxicology studies of both derivatives demonstrated considerable cardiovascular safety risk in normal rats, arising from significant inhibition of hERG K^+^ channels (IC_50_ in the range 37–463 nM). For this reason, in the attempt to reduce the off-target effect, the same researcher group developed a new set of quinoline molecules, among which the compounds **24** and **25** were identified as the most active, (Figure 7a). The modification was directed towards the C-3 piperazinyl portion with the aim of improve the PK profile and reduce the hERG activity without influencing the key interactions. In detail, bearing a Michael acceptor moiety to the N-4 piperazinyl ring, these new quinoline compounds showed c-Met IC_50_ values comparable to the parent molecules (2.3 and 2.6 nM respectively for **24** and **25**), and an appreciable lower activity against hERG [57]. 

**Figure 8:**
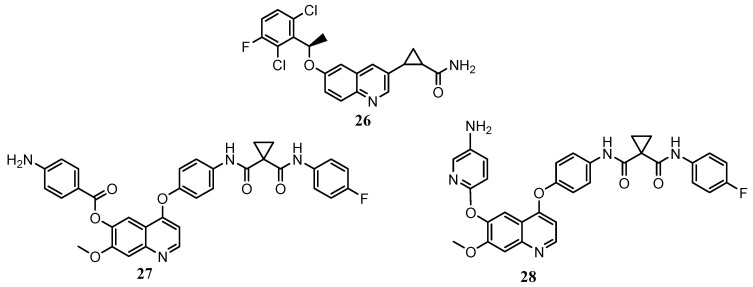
4,6,7-substitued quinoline derivatives selectively active on c-Met [58,59].

Nishii and co-workers explored modifications at the C-3 and C-6 positions of quinoline scaffold. 3,6-disubstituted quinoline **26** (Figure 8), shows an inhibition effect selectivity against c-Met kinase among more than 20 kinases with IC_50_ = 9.3 nM, and a potent antiproliferative activity against MKN45 cancer cell line (IC_50_ = 0.093 μM), [58].

**Figure 9:**
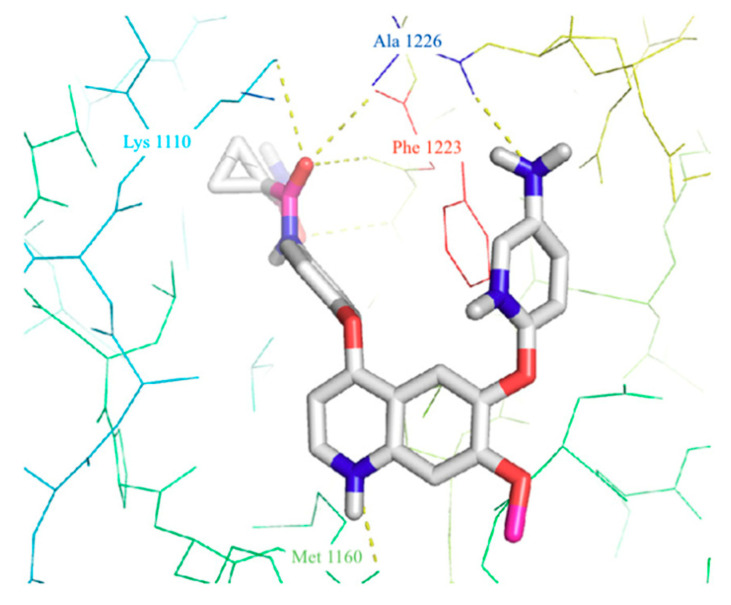
Main interactions of compound **28** with the amino acid residues in c-Met active site [59].

In 2019, focusing the attention on C-6 substitution, Lien and co-workers designed and evaluated several 4,6,7-substituted quinolines, analogues of cabozantinib (Figure 8) [59]. In vitro antiproliferative assays highlight that derivatives **27** and **28** are more active than the parent compound on leukaemia, CNS, and breast cancer cell lines. The c-Met kinase inhibition assay denotes higher, or comparable activity than cabozantinib, with IC_50_ values of 19 and 64 nM respectively for **27** and **28** (IC_50_ cabozantinib = 40 nM). On this basis, from the point of view of structure–activity relationships, these derivatives demonstrated well fittings in the binding pocket of c-Met. Indeed, docking studies (Figure 9) demonstrated that, besides the interactions with crucial amino acids residues, above observed, these compounds could engage, through the p-aminophenyl moiety at C-6, additional hydrogen bonding with Ala^1226^ [59]. 

**Figure 10:**
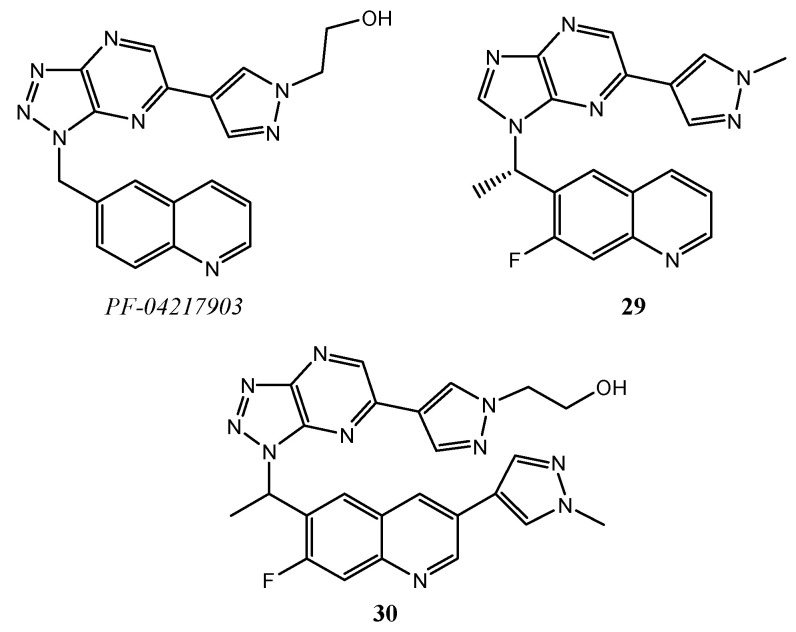
Structures of some hybrid quinoline derivatives as c-Met inhibitors.

Different studies on hybrid derivatives, with a quinoline scaffold linked at the C-6 position to another heterocyclic system, as triazolo-pyrazine or imidazo-pyrazine, demonstrate inhibition activity on c-Met and antiproliferative effects unless the 4-phenoxy substitution typical of the cabozantinib/foretinib-like compounds. In detail, Cui and co-workers described anticancer properties of 2-(4-(1-(quinolin-6-ylmethyl)-1H-[1,2,3]triazolo[4–*b*]pyrazin-6-yl)-1H-pyrazol-1-yl)-ethanol methanesulfonate (PF-04217903), (Figure 10), an extremely potent and selective c-Met inhibitor with good oral bioavailability and an acceptable safety profile in preclinical trials [60].

After SGX523 and JNJ-38877605 clinical failure [61,62,63], Zhang and co-authors synthetized 3-N-substituted quinoline triazolopyridine analogues to understand the electron-donating and steric hindrance effects on the metabolic activity of the aldehyde oxidase [64]. 

Furthermore, Zhao et al. have conducted some studies on both triazolo[4,5-*b*]pyrazine and imidazo[4,5-*b*]pyrazine quinoline derivatives **29**, **30** (Figure 10), showing significant inhibitory effects on both enzymatic and cellular assay, as well as in vivo selectivity and metabolic stability [65,66]. 

### 2.2. Quinoline as Inhibitor of PI3k/AkT/mTOR Pathway 

A lot of quinoline derivatives, basically substituted or condensed with other ring systems, exhibit inhibitory activity on PI3K/AkT/mTOR pathway and several molecules are currently in clinical trials. For example, omipalisib (GSK2126458) is a quinoline derivative, substituted at C-6 with a 2-(methoxy)-3-benzenesulfonamide pyridinyl moiety, at present under investigation in the first-in-human phase I study. This molecule shows the high inhibition effect of both PI3K and mTOR with an acceptable oral bioavailability [67,68]. Several omipalisib-like derivatives, characterized by a N-{5-[6-quinolinyl]-3-pyridinyl}benzenesulfonamide scaffold, have been synthesized and biologically evaluated for their PI3K/mTOR inhibition capability (biological data of omipalisib and compounds **31**–**34** are reported in Table 2) [69,70,71,72].

**Figure 11:**
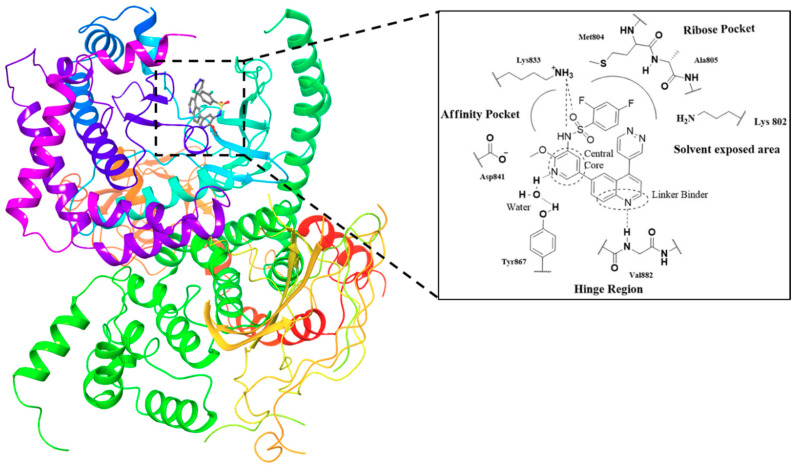
Crystal structure of *omipalisib* in complex with the catalytic subunit of PI3Kγ (PDB id: 3L08) and detailed interactions [67,73].

Crystallography and in silico studies highlight some recurrent and crucial interactions between these ligands and the ATP-binding site of PI3Kγ: the backbone NH of Val^882^ (Val^851^ in PI3Kα) forms a hydrogen bond with quinoline nitrogen; Lys^883^ interacts, through a charged bond, with the sulfonamide group; and the C-4 quinoline substituent frequently stabilizes the ligand-protein complex, accommodating itself in the ribose pocket physiologically occupied by ATP. In Figure 11 it is reported the co-crystal structure of omipalisib in complex with the catalytic subunit of PI3Kγ, [67,69,70,71,72].

Ma and co-workers reported the synthesis and the biological activities of a series of quinoline derivatives with a substituted aniline at C-4 position, and linked to another quinoline moiety at the C-6 one (compound **35**–**37** in Table 3). These quinoline compounds showed interesting inhibitory activities on both mTORC1 and mTORC2, with an adequate stability in simulated gastric, intestinal fluids, and liver microsome. The docking studies for these derivatives into the mTOR catalytic cleft showed the importance of some recurrent interactions, that stabilize the complex: hydrogen bond between the nitrogen of central quinoline scaffold and the critical residue Val^2240^, π– π interaction between indole moiety of Trp^2239^ and quinoline scaffold, and hydrogen bond between quinoline substituent at C-6 and Tyr^2225^ [74,75].

**Figure 12:**
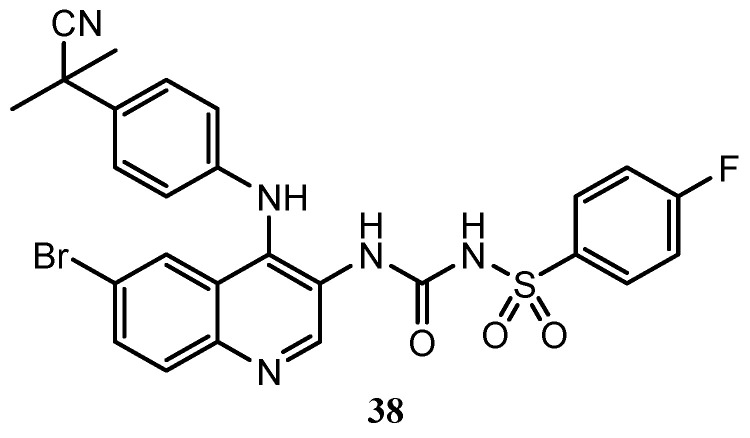
The most active derivative of 3-phenylsulfonylurea-4-aniline quinoline series [76].

Zhao et al. published the synthesis and the biological activity of quinoline derivatives with dual PI3K/mTOR inhibitory effects. This new set of 4-aniline quinoline compounds containing phenylsulfonylurea are a combination of the benzensulfonamido and the anilino moieties, which are generally present in most of the quinoline active molecules reported in this review. Quinoline **38** (Figure 12) is the best inhibitor of the series, with IC_50_ values against PI3K and mTOR, respectively of 0.72 μM and 2.62 μM, and with an IC_50_ (half-maximal inhibitory concentration) against MCF-7 cell line comparable to the positive control [76]. 

**Figure 13:**
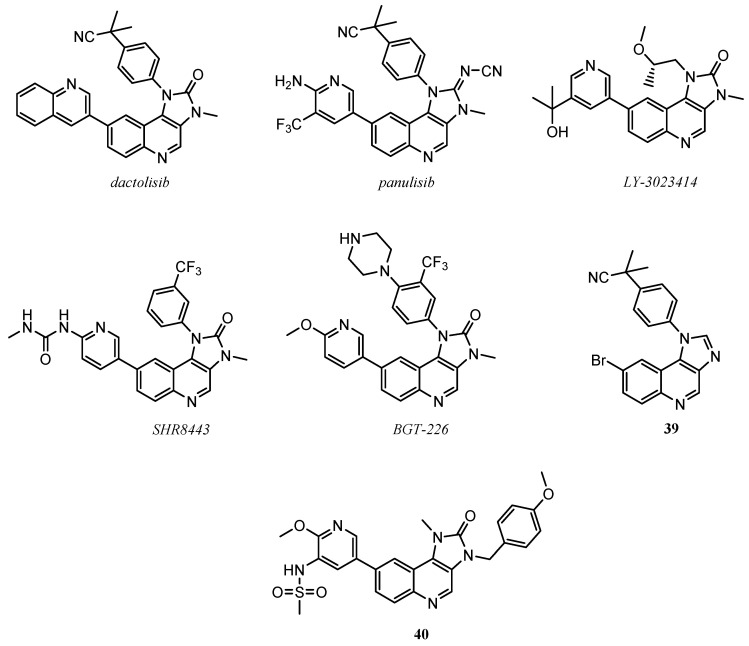
Imidazo[4,5-*c*]quinoline derivatives as promising anticancer PI3K/mTOR inhibitors.

The PI3K/mTOR inhibition activity is also conserved in numerous derivatives in which the quinoline scaffold is condensed with other heterocycles. Among these, imidazo[4,5-*c*]quinolines are the most important, such as investigational drugs like dactolisib (BEZ-235), panulisib (P-7170), LY-3023414, SHR8443, and BGT-226 (Figure 13) [3]. 

**Figure 14:**
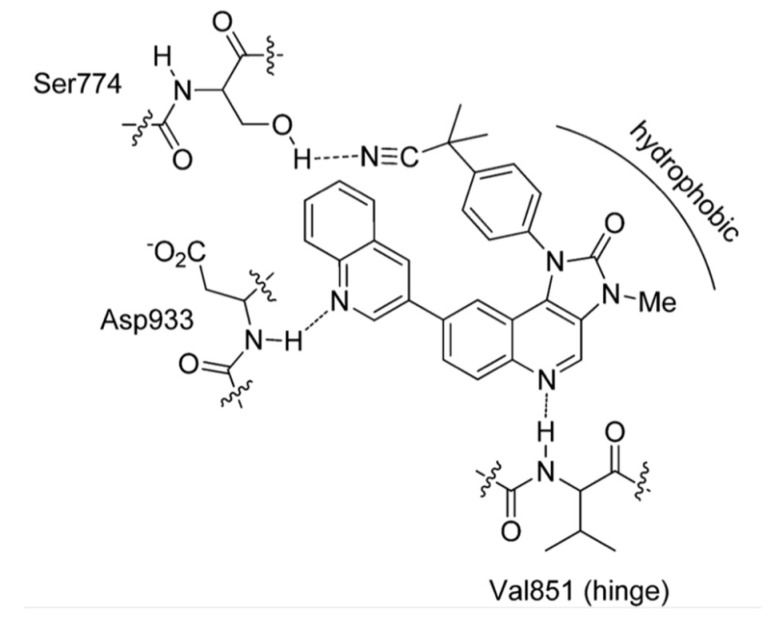
Model for the interactions of *dactolisib* in the ATP-binding cleft of PI3Kα [78,84].

Dactolisib,2-methyl-2-[4-(3-methyl-2-oxo-8-quinolin-3-yl-2,3-dihydro-imidazo[4,5-*c*]quinolin-1- -yl)-phenyl]-propionitrile, was patented by Novartis in 2006 [77]. It demonstrated high selectivity and potency in the nanomolar concentration range against PI3K/mTOR kinases, acceptable oral bioavailability, potent antiproliferative activity on acute lymphoblastic leukaemia T-cell and also in paclitaxel-resistant gastric cancer models. Moreover, it has been tested in clinical trials, alone or in combination with cytotoxic agents, in patients with advanced solid tumours, as for example nasopharyngeal carcinoma [78,79,80,81,82,83]. From a molecular point of view, in Figure it 14 is shown the binding mode of dactolisib in the catalytic site of PI3Kα as an example for all the imidazo[4,5-*c*]quinolines: the nitrogen of the quinoline core forms an hydrogen bond with the backbone of the conserved Val^851^, interaction observed also for the ATP; other two hydrogen bonds involved the -CN substituent and the nitrogen of the lateral quinoline scaffold respectively with the amino acids Ser^774^ and Asp^933^ (Figure 14) [78,84].

Panulisib (P-7170) was patented in 2012 [85]. This quinoline compounds demonstrated potent inhibition activity on mTORC1/mTORC2/ALK1, cell cycle arrest, induction of apoptosis, antiangiogenic activity. Moreover, it showed antitumor efficacy on both in vitro and in vivo studies against the erlotinib–sensitive and –insensitive models NSCLC and anti-estrogen models of ER+ breast cancer [86,87,88], reaching the clinical trials evaluation for patients with advanced refractory solid tumours (ClinicalTrials.gov number NCT01762410) [89]. 

LY-3023414, developed as a selective PI3K/mTOR dual inhibitor, was patented in 2012, with an absolute mTOR IC_50_ and PI3Kα IC_50_ values respectively of 0.165 μM and 0.00607 μM. In cancer cell human line panels, it showed broad antiproliferative activity [90,91]. In a first-in-human phase I study it demonstrated a tolerable safety profile and single-agent activity in patients with advanced tumours [92]. 

SHR8443 has been evaluated in vitro for the enzymatic inhibition assay of mTOR, showing IC_50_ value of 1 nM [93]. Preclinical studies evidenced pharmaceutical properties favourable for the clinical use in antitumor treatments [94]. 

BGT-226, 8-(6-Methoxy-pyridin-3-yl)-3-methyl-1-(4-piperazin-1-yl-3-trifluoromethyl-phenyl)- 1,3-dihydro-imidazo[4,5-*c*]quinolin-2-one, developed by Novartis [77], demonstrated effectiveness in reducing in vitro and in vivo the growth of several human cancer types: head and neck [95]; human pancreatic [96]; human acute leukaemia and myeloma cancer cell lines [97,98]; NSCLC cancer (in combination with gefitinib) [99]; normoxic and hypoxic hepatocarcinoma cells [100]. BGT-226 reached the phase I of clinical trials (ClinicalTrials.gov Identifier: NCT00742105) where it demonstrated good tolerability, but exhibited insufficient results for a clinically meaningful PI3K pathway inhibition [101].

Recently, new series of imidazo[4,5-*c*]quinoline derivatives have been reported in the literature as PI3K/mTOR inhibitors. Xiao et al. proved anticancer properties of a set of quinoline molecules, from which compound **39** (Figure 13) is the most active, with a mTOR IC_50_ value of 1.4 μM and PI3Kα IC_50_ of 0.9 μM [102]. 

Bahekar et al. synthesized quinoline **40**, an orally available, selective, and effective inhibitor of PI3Kδ, with IC_50_ value of 1.9 nM [103]. 

**Figure 15:**
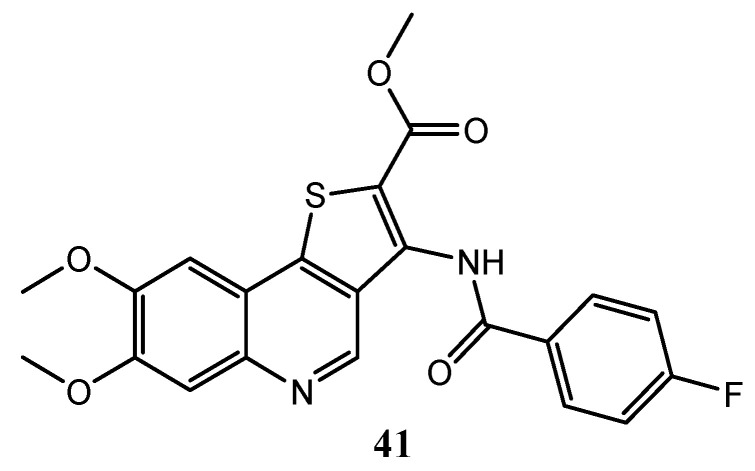
Thieno[3,2-*c*]quinoline compound active as PI3K inhibitor [104].

Besides imidazo[4,5-*c*]quinolines, also some interesting thieno[3,2-*c*]quinoline derivatives show PI3K inhibitory activity; compound **41** (Figure 15), for example, exhibits a PI3K IC_50_ = 1 μM and IC_50_ values against K562 and DU145 cancer cell lines of 0.15 μM and 2.5 μM, respectively [104]. 

**Figure 16:**
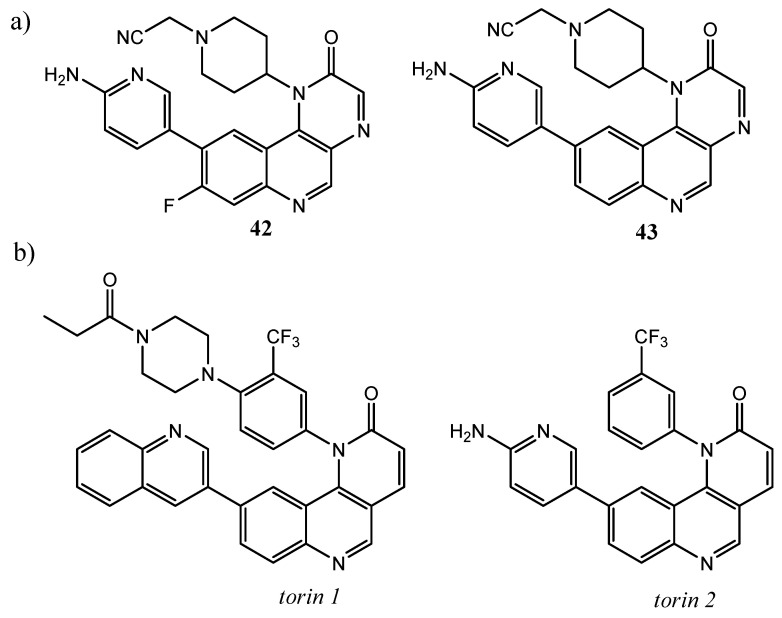
(**a**) mTOR inhibitors containing pyrazino[2,3-*c*]quinolin-2(1H)-one scaffold; (**b**) structures of *torin 1* and *torin 2*, mTOR inhibitors containing benzo[*h*]-1,6-naphthyridin-2(1H)-one scaffold [105,106,107].

Furthermore, recently, Guo et al. through high-throughput screening (HTS), selected a novel hit compound **42** (Figure 16a), containing pyrazino[2,3-*c*]quinolin-2(1H)-one scaffold, as mTOR inhibitor. This molecule demonstrated an mTOR IC_50_ = 31 nM, but weak antiproliferative activity in vitro. Further structural optimization led to compound **43** (Figure 16), that showed a better mTOR inhibitory activity than the parent compound (IC_50_ = 7 nM), good selectivity profile, potent antiproliferative activities against breast and cervical cancer lines, and significant tumor regression in the T-47D xenograft model after an oral once-daily dose [105]. 

Remarkable interesting, are torin 1 and torin *2*, two benzo[*h*][1,6]-naphthyridin-2(1*H*)-one compounds, containing a quinoline moiety and acting as selective mTOR inhibitors (Figure 16b). Developed by Liu and co-workers, these tricyclic pyridoquinolines derivatives are the result of two consecutive generations of quinoline-based compounds. They are active on the phosphorylation process of mTORC1/ mTORC2 substrates in the cell, exhibiting EC_50_ values in the range 0.25–7 nM. Furthermore, with respect to torin 1, second generation compound torin 2 demonstrates 800-fold selectivity over PI3K, improving the bioavailability (54%), metabolic stability, and plasma exposure [106,107]. 

### 2.3. Quinoline Derivatives as Inhibitors of Epidermal Growth Factor Receptors 

**Figure 17:**
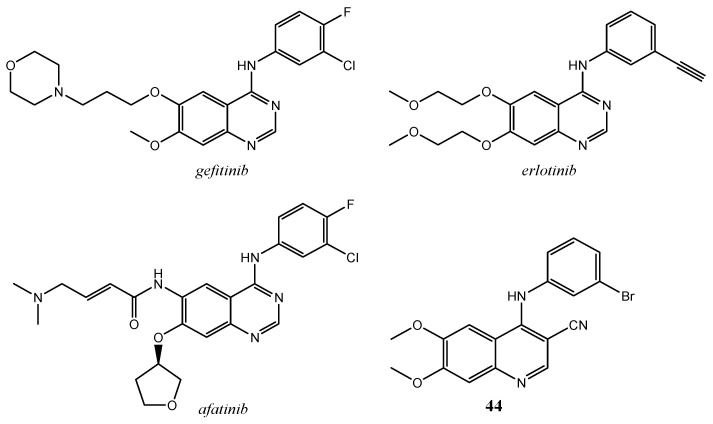
4-anilinoquinoline-3-carbonitrile **44** and of the approved 4-anilinoquinazoline EGFR inhibitors *gefitinib, erlotinib, afatinib*.

Various quinoline derivatives, with EGFR inhibitory potential, have been studied and the 4-anilinoquinoline-3-carbonitrile class is one of the first group developed. These compounds have been designed by molecular modelling studies, considering the approved EGFR 4-anilinoquinazoline inhibitors (gefitinib, erlotinib, and afatinib) as lead compounds (Figure 17). 

Weissner et al. demonstrated that the N-3 of quinazoline ring system can be replaced by a C-X, where X is an electron withdrawing group (i.e., CN). It was found that 4-anilinoquinoline-3-carbonitriles are effective inhibitors of EGFR kinase with activity comparable to the 4-anilinoquinazoline-based inhibitors. Compound **44** (Figure 17) showed the interesting IC_50_ value of 7.5 nM [108].

**Figure 18:**
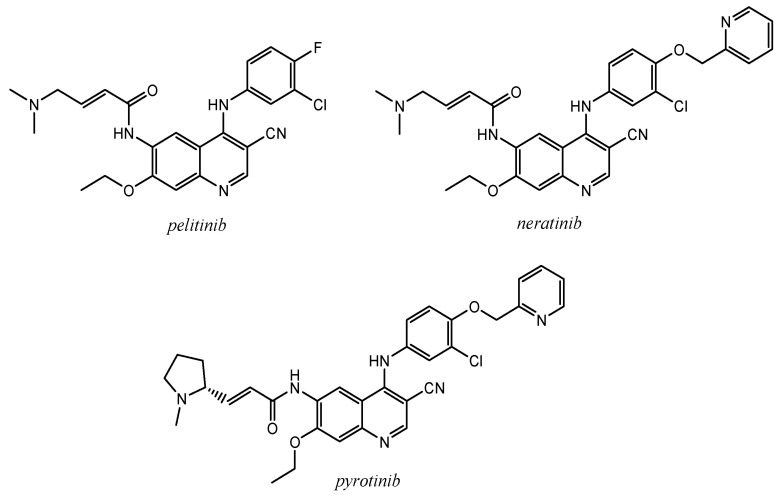
Structures of the irreversible EGFR inhibitors *pelitinib* (EKB-569), *neratinib* (HKI-272), and *pyrotinib* (SHR-1258).

The same authors developed a second generation of EGFR inhibitors, namely 4-anilinoquinoline-3-carbonitrile derivatives bearing Michael acceptor groups (such as 4-(dimethylamino)crotonamide) at the position C-6 (Figure 18).

These molecules act as EGFR irreversible inhibitors, forming a covalent bond with a conserved cysteine residue located in the ATP binding pocket of EGFR (Cys^773^ or Cys^797^) and HER-2 (Cys^805^) [109]. In particular, pelitinib (EKB-569; EGFR IC_50_ = 0.083 μM), currently under investigation in clinical trials, showed excellent oral in vivo activity [3,110].

**Figure 19:**
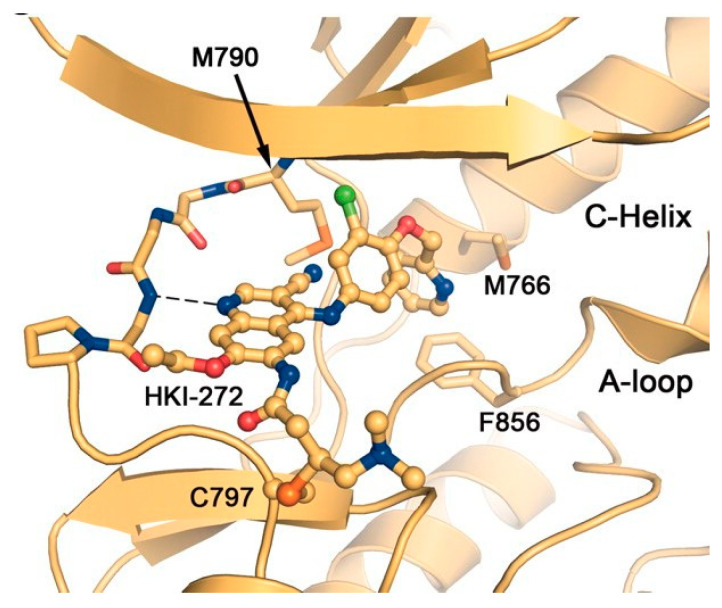
EGFR/T290M mutant kinase domain (PDB id: 2JIV) in complex with *neratinib* [113].

Subsequently, Tsou et al., introducing a lipophilic substituent at the para position of the 4-arylamino ring, isolated the molecule HKI-272, more active than pelitinib, and approved in 2017 with the name neratinib (Figure 18) for the extended adjuvant therapy against early-stage HER2/ErbB2-amplified/overexpressed breast cancer [3,4,109,111,112]. Crystal structure of EGFR/T290M mutant kinase domain in complex with neratinib showed the main interactions recurrent in the binding pocket (Figure 19): non-covalent interactions (H-bond of quinoline nitrogen with residues of the hinge region; hydrophobic interactions between the 2-pyridinyl group and the Met^766^, Phe^856^ and Met^790^) and a covalent bond (Cys^797^ at the edge of the active site cleft attacked the crotonamide group of the ligand) [113].

**Figure 20:**
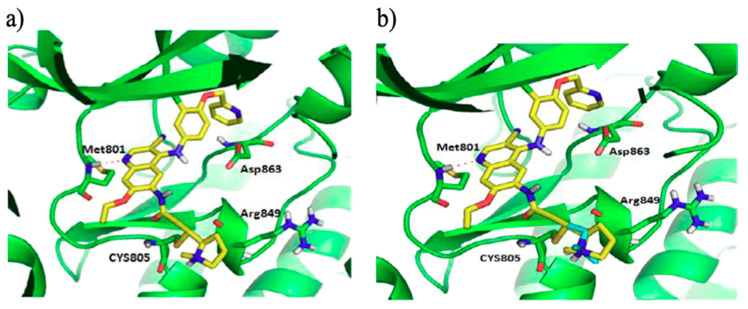
(**a**) binding mode of *pyrotinib* in the catalytic region of HER2 kinase; (**b**) overlap binding mode of *neratinib* (colored blue) and *pyrotinib* (colored yellow) [114].

Starting from the lead compound neratinib, SHR1258 has been developed as irreversible EGFR/HER2 tyrosine kinase inhibitor, and afterwards named pyrotinib (Figure 18). Figure 20 shows a comparison between binding modes of neratinib and pyrotinib in the catalytic region of HER2 kinase. In this model the two molecules are completely superimposed (Figure 20b), except for the substituents on the Michael acceptor group. Both display the same stabilizing interactions: hydrogen bond between N-1 of the quinoline ring and the hinge region of Met^801^, a covalent bond between Cys^805^ and Michael acceptor group (double bond) [114]. 

In vitro preclinical studies show high potency of pyrotinib, comparable to neratinib, against HER2 dependent BT474 (breast cancer) and SK-OV-3 (ovarian cancer) cell lines and also in vivo efficacy in HER2-dependent mouse xenograft models. Moreover, pyrotinib displays high selectivity when tested against a panel of different kinases [114]. Clinical trials demonstrate a safety profiles, desirable pharmacokinetic properties, tolerability and promising antitumor activity in HER2-positive patients with metastatic breast cancer, especially in combination with capecitabine [115,116]. Currently, pyrotinib is also under trial evaluation for the use in HER2-positive gastric cancer and NSCLC [117].

**Figure 21:**
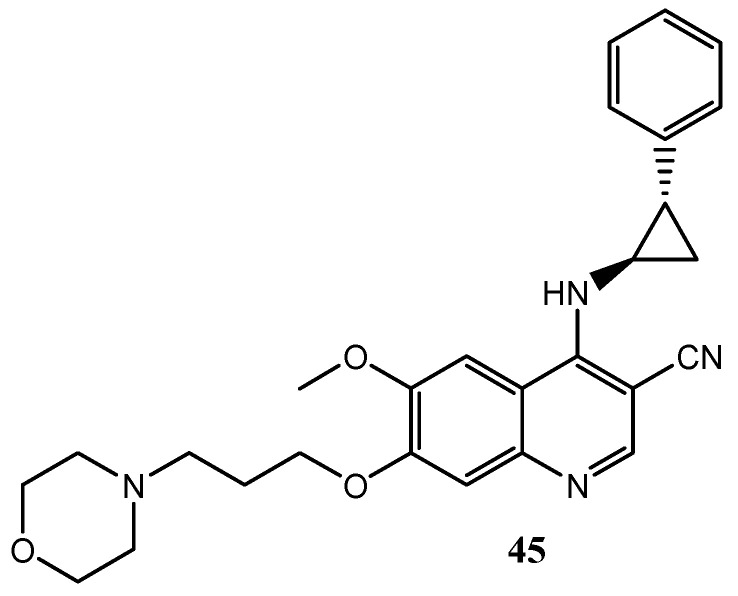
Structure of 4-(2-aryl-cyclopropylamino)-quinoline-3-carbonitrile as selective inhibitor of EGFR [118].

Pannala et al. described the synthesis and the evaluation of EGFR inhibition of some 4-(2-aryl-cyclopropylamino)-quinoline-3-carbonitriles. These compounds possess an arylcyclopropylamino group at the C-4 position of the quinoline-3-carbonitrile core instead of 4-aniline one. Quinoline **45** (Figure 21) shows an interesting inhibition effect on EGFR with IC_50_ value of 5 nM [118]. 

**Figure 22:**
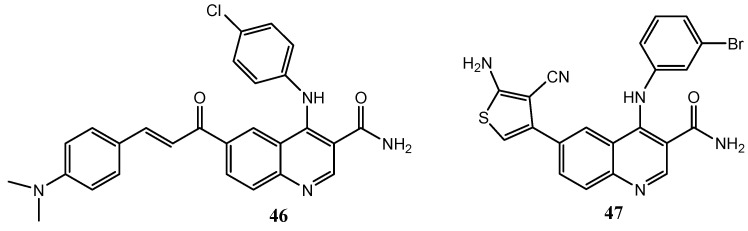
4-anilino-3-carboxyamide derivatives as EGFR inhibitors [119,120].

Aly et al. planned and synthesized a set of 4-anilino-3-carboxyamide derivatives. They based their studies on the high similarities between the 4-anilinoquinazoline, 4-anilinoquinoline-3-carbonitrile, and 4-anilinoquinoline-3-carboxamide nuclei. In a previous work, quinoline **46** (EGFR IC_50_ = 5.283 μM) was selected as a lead structure for the design of other more active derivatives [119]. In particular, compound **47** with a substituted-thiophene moiety at C-6 (Figure 22) exhibited selective activity on EGFR with IC_50_ value of 0.49 μM [120]. 

**Figure 23:**
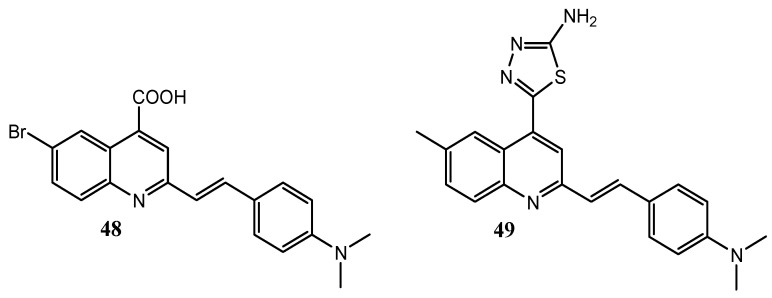
2-styrylquinolines **48** and **49** as promising EGFR inhibitors [121].

In addition, 2-styrylquinolines **48** and **49**, without a 4-anilino substitution (Figure 23), have been synthesized by El-Sayed et al., showing moderate inhibition effects towards EGFR with IC_50_ values of 2.23 and 1.11 μM respectively [121]. 

**Figure 24:**
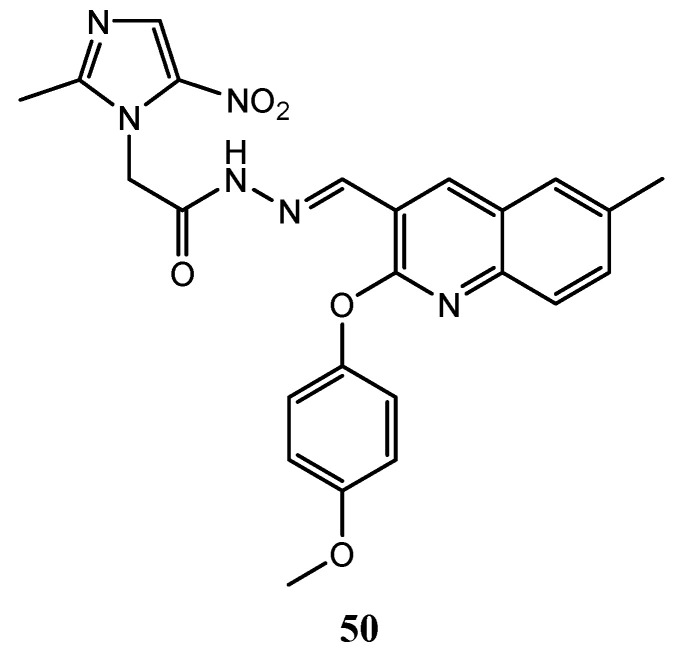
Schiff’s base quinoline derivative as EGFR tyrosine kinase inhibitor [122].

In lasts years, several hybrids derivatives containing quinoline scaffold linked with other heterocyclic ring systems have been studied as EGFR inhibitors with interesting activity. Makawana et al. reported numerous Schiff’s base derivatives bearing nitroimidazole and quinoline nuclei as potential EGFR tyrosine kinase inhibitors. Compound **50** (Figure 24) showed an IC_50_ value on EGFR receptor of 0.12 ± 0.05 μM [122]. 

**Figure 25:**
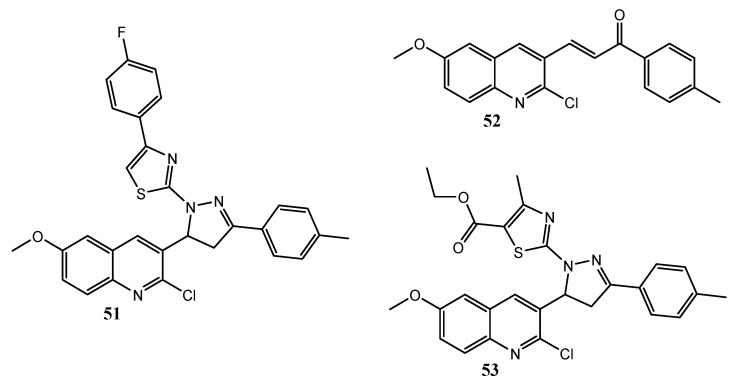
Pyrazoline/pirazolinylthiazole quinoline hybrids [123].

George et al. described some new quinoline hybrid derivatives bearing either pyrazoline or pirazolinylthiazole heterocycles. Most of the tested compounds revealed promising anticancer activity especially against DLD1 cancer cell line (colorectal). Furthermore, compounds **51**–**53** (Figure 25) revealed inhibitory effect on EGFR at nanomolar concentration, with IC_50_ values of 31.80, 37.07, and 42.52 nM, respectively [123].

The quinoline scaffold is frequently present in numerous polycondensed ring system compounds with proved selective inhibition activity on EGFR.

**Figure 26:**
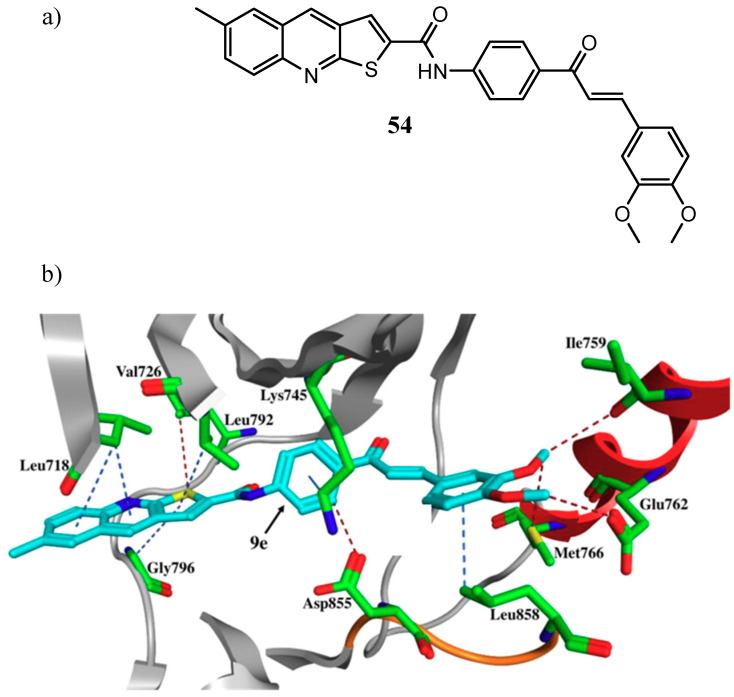
(**a**) Thieno[2,3-*b*]quinoline-2-carboxamide-chalcone derivative **54**; (**b**) orientation and interactions of **54** within the EGFR active site [124].

Abdelbaset et al., for example, designed several thieno[2,3-*b*]quinoline-2-carboxamide-chalcone derivatives, as molecule **54** (Figure 26a) that showed significant antiproliferative effects against tested cancer cell lines (IC_50_ values in the range 0.9–1.2 μM) and an EGFR IC_50_ = 0.5 μM. Molecular docking studies showed that the thienoquinoline moiety occupied the ATP-binding site of the receptor, whereas chalcone moiety was located in an allosteric pocket of the enzyme (Figure 26b) [124]. 

**Figure 27:**
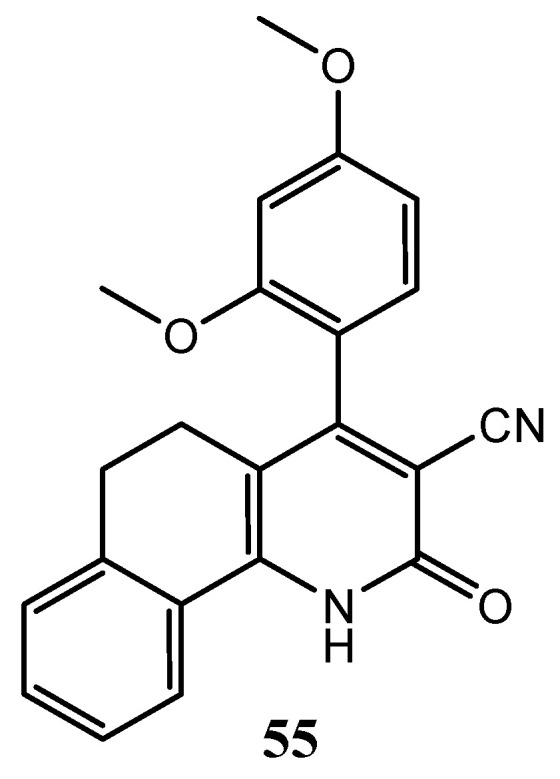
Structure of benzo[*h*]quinoline derivative [125].

Afterwards, Abdelsalam and co-workers designed some benzo[*h*]quinoline derivatives, as molecule **55** (Figure 27), with good anticancer activity against MCF-7 cancer cell line (IC_50_ = 7.21 ± 0.43 μM) and also an EGFR IC_50_ = 0.14 μM [125]. 

### 2.4. Quinoline Derivatives as Inhibitors of Vascular Endothelial Growth Factor Receptor

VEGF-VEGFR abnormal signals play central roles in angiogenic processes in a variety of diseases, especially in cancer. Some studies demonstrated, that, although the VEGF binds more effectively the VEGFR-1, its major mitogenic and angiogenic effects seem to be mediated through the interaction with VEGFR of type 2. In recent years, a lot of selective angiogenesis and multi-receptor kinase inhibitors have been synthesized, and some of these have been approved for clinical use [24,25].

**Figure 28:**
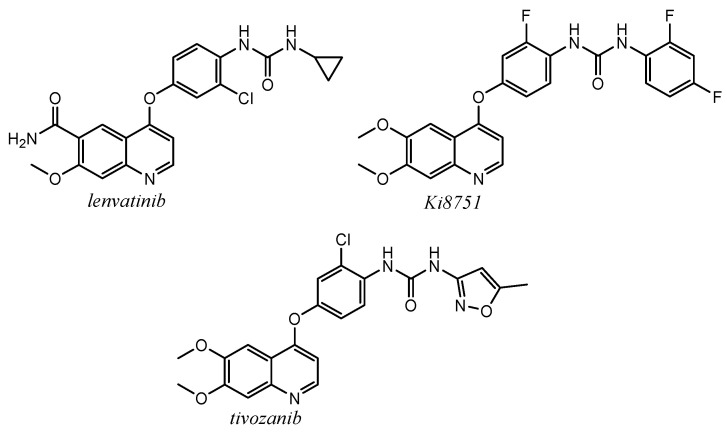
Structures of VEGFR inhibitors with quinoline-urea moiety *lenvatinib*, *Ki8751*, *tivozanib*.

Lenvatinib, (Figure 28), approved in 2015 for the treatment of differentiated thyroid, hepatocellular cancers, and the second-line treatment of renal carcinoma, is a multikinase quinoline inhibitor with a selective effect against VEGFR [4,7]. 

Many other quinoline derivatives are reported to possess selective VEGFR inhibitor activity, especially against VEGFR-2. Kubo et al. reported the synthesis of some N-Phenyl-N’-{4-(4-quinolyloxy)phenyl}ureas, such as compound Ki8751 (Figure 28), which exhibited the best inhibition effect on VEGFR with IC_50_ value of 0.9 nM [126]. 

Nakamura and co-workers firstly reported the antitumor and anti-angiogenic activity of KEN951, after called tivozanib, (Figure 28). With a very similar structure to the previous studied compounds, this quinoline-urea inhibits VEGFR-1, VEGFR-2, and VEGFR-3 tyrosine kinases (IC_50_ = 30, 6.5, and 15 nM, respectively). Tivozanib potently inhibited VEGF-induced VEGFR-2 phosphorylation in endothelial cells at in vitro sub-nanomolar concentration (IC_50_ = 0.16 nM). In xenografts models, tivozanib demonstrated significant inhibition of tumor-induced angiogenesis and tumor vessel normalization and in phase I of clinical trial it exhibited safety and tolerability when orally administered [127,128,129].

**Figure 29:**
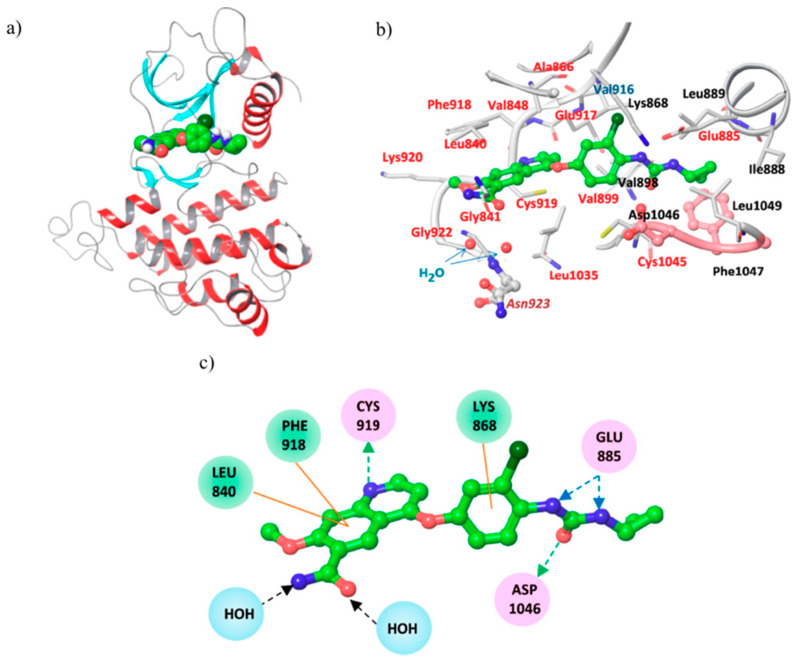
(**a**) crystal structure of *lenvatinib* in complex with VEGFR-2 (PDB id: 3WZD); (**b**) binding pocket of VEGFR-2 occupied by *lenvatinib*; (**c**) scheme for interactions of *lenvatinib* in the VEGFR-2 binding pocket [130].

Analysing the structural analogies between lenvatinib, Ki8751, and tivozanib, it is possible to underline the recurrent and important presence of the C-4 oxyphenyl urea substituted moieties and the C-6, C-7 substitutions on the quinoline scaffold. These structure features play a pivotal role for the stabilization of ligand-protein complex, as it is shown by the X-ray crystal structure of VEGFR-2 in complex with lenvatinib (Figure 29, PDB id: 3WZD): the ligand binds the receptor kinase domain in its DFG-in conformation, occupying the ATP-binding site through its quinoline moiety and the neighboring region via the cyclopropane ring. In particular, the key interactions are three H-bonds (quinoline nitrogen and Cys^919^; urea oxygen and Asp^1046^; urea nitrogen and Glu^885^), two hydrophilic interactions (the C-6 carboxamide with Asn^923^, bridged by water molecules) and several π-interactions (quinoline and C-4 phenoxy moiety with Leu^840^, Phe^818^ and Lys^868^) [130]. Quinoline core and urea moiety in tivozanib showed the same key interactions observed for the parent compound (PDB id: 4ASE) [131,132].

**Figure 30:**
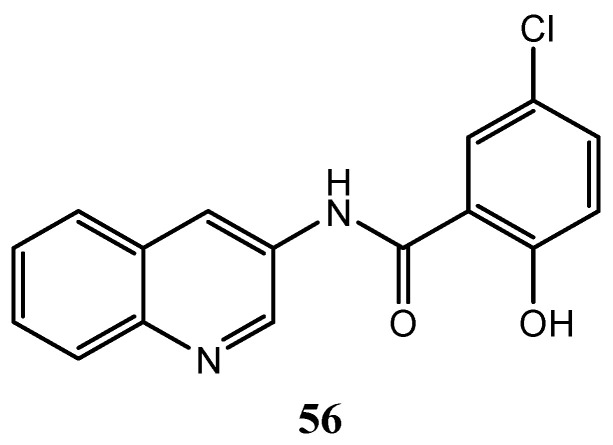
Structure of the quinoline VEGFR-2 inhibitor **56** [133].

Yang et al. reported the synthesis of some quinoline amide derivatives with VEGFR-2 inhibition activity. Compound **56** (Figure 30) resulted to be the most active to inhibit VEGFR kinase (IC_50_ = 3.8 nM) and proliferation of HUEVEC cancer cells (IC_50_ = 5.5 nM); the docking analysis confirmed that this compound is suitable bonded to VEGFR-2 [133]. 

**Figure 31:**
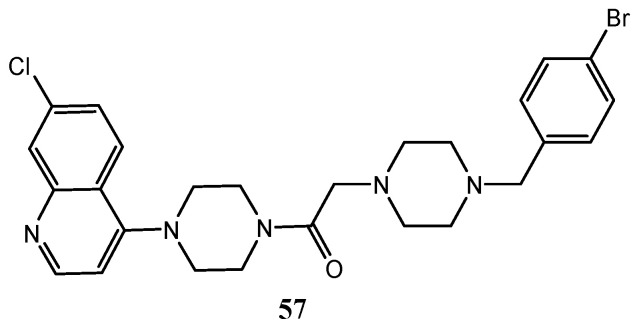
Structure of compound **57**, a 7-Chloro-4-(piperazin-1-yl)quinoline derivative with VEGFR-2 inhibition activity [134].

Through a combination of some structural elements present in molecules with antiproliferative activity, such as 4-piperazinoquinoline scaffold and aminoacyl chain, Aboul-Enein et al. planned and synthesized 7-Chloro-4-(piperazin-1-yl)quinoline derivatives as VEGFR-2 inhibitors. The most promising compound **57** (Figure 31), exhibited cytotoxicity higher than that of reference drug (doxorubicin) against MCF-7 cell line (6.502 μM vs. 6.774 μM) and a VEGFR-2 IC_50_ = 1.38 μM, but less potent than the reference drug sorafenib (IC_50_ of 0.33 μM). Docking analysis in the ATP-binding site of VEGFR-2 proved that molecule **57** shows a binding mode similar to that of other VEGFR-2 inhibitors as lenvatinib: both displayed H-bonds between quinoline nitrogen and Cys^919^, and between the carbonyl group (in the aminoacyl and in urea moieties in derivative **57** and tivozanib, respectively) and Asp^1046^. However, **57** showed some additional π-interaction with Ile^888^ and Phe^918^ [134]. 

**Figure 32:**
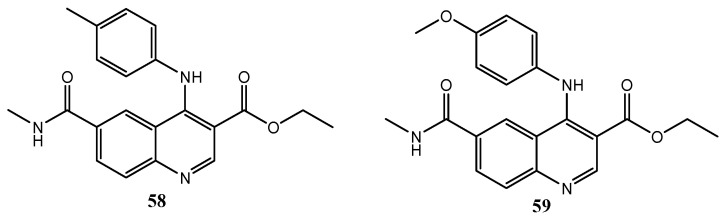
Quinoline-3-carboxylic acid derivatives with VEGFR-2 and VEGFR-3 inhibition activity [135].

In 2008, Westman et al. published a series of quinoline-3-carboxylic acid derivatives (Figure 32), acting as VEGFR-2 and -3 inhibitors and with good potency and high selectivity for the treatment and prevention of cell proliferative and/or differentiation disorders. Compounds **58** and **59** showed IC_50_ values against VEGFR-2 of 1.2 μM and 2.5 μM, and against VEGFR-3 of 0.53 μM and 0.35 μM respectively [135].

**Figure 33:**
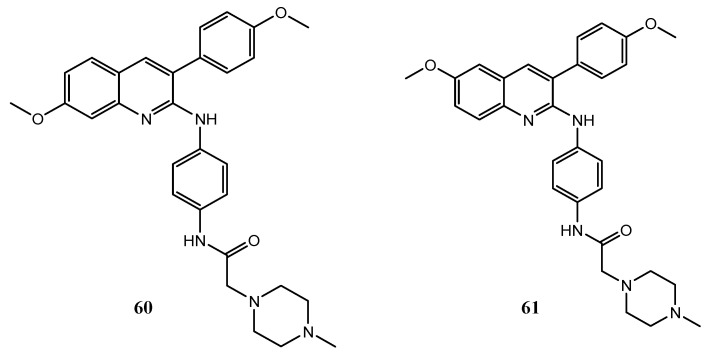
Structure of 3-aryl-quinoline derivatives **60** and **61**, dual VEGFR-2/ERα inhibitors [136].

Different quinoline derivatives are reported to act as dual inhibitors of VEGFR and other targets involved in carcinogenic processes. For example, Li et al. discovered some 3-aryl-quinoline derivatives **60** and **61** as dual inhibitors of both VEGFR-2 and ERα. Two molecules (Figure 33) showed interesting IC_50_ against ERα and VEGFR compared to the reference compounds, tamoxifen and sunitinib, respectively (for compound **60**, ERα IC_50_ = 2.33 μM and VEGFR-2 IC_50_ = 104 nM; for **61**, ERα IC_50_ = 1.78 μM and VEGFR-2 IC_50_ = 86 nM) [136]. 

### 2.5. Quinoline Derivatives as Inhibitors of Ras/Raf/MEK Pathway 

EGFR and VEGFR extracellular activations trigger the intracellular cascade of signalling connected to Ras/Raf/MEK/ERK. This deregulated pathway, in neoplastic cells, improve the tumour genesis, acting on the survival processes of cellular proliferation; such as differentiation, apoptosis, angiogenesis.

**Figure 34:**
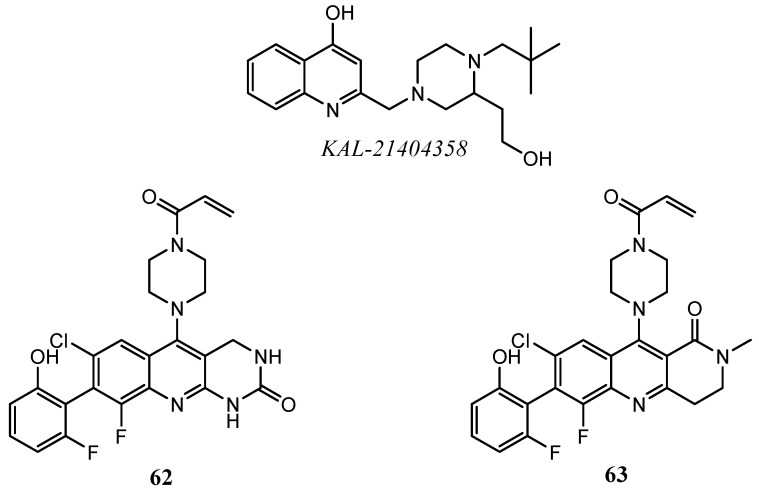
Quinoline-based molecules with inhibition activity on Ras.

Several quinoline derivatives, tested as inhibitors of Ras/Raf/MEK cascade, demonstrate promising results both in antiproliferative and enzymatic inhibition assays. In detail, Feng and co-workers developed quinoline KAL-21404358 (Figure 34), the first ligand of the K-Ras P110 allosteric pocket able to disrupt downstream pathways (Raf/MEK/ERK and PI3K/AkT/mTOR). Through a combination of computational methods and with the validation of biochemical assays, the authors proposed a mechanism of action for KAL-21404358. The quinoline compound could interfere with the protein-protein interactions, binding and stabilizing K-Ras in its inactive GDP-bound state, then, it halts the nucleotide exchange process and the subsequent activation of Ras [137].

In 2016, Li et al. filled a patent of a new set of fused-tricyclic compounds containing quinoline scaffold with a promising inhibition activity on the mutated form of Gly12Cys K-Ras (Figure 34). The analysis of the catalytic binding site, highlights that Cys^12^ forms a covalent bond with the electrophilic acryloyl moiety of quinolines **62** and **63 [138]**.

**Figure 35:**
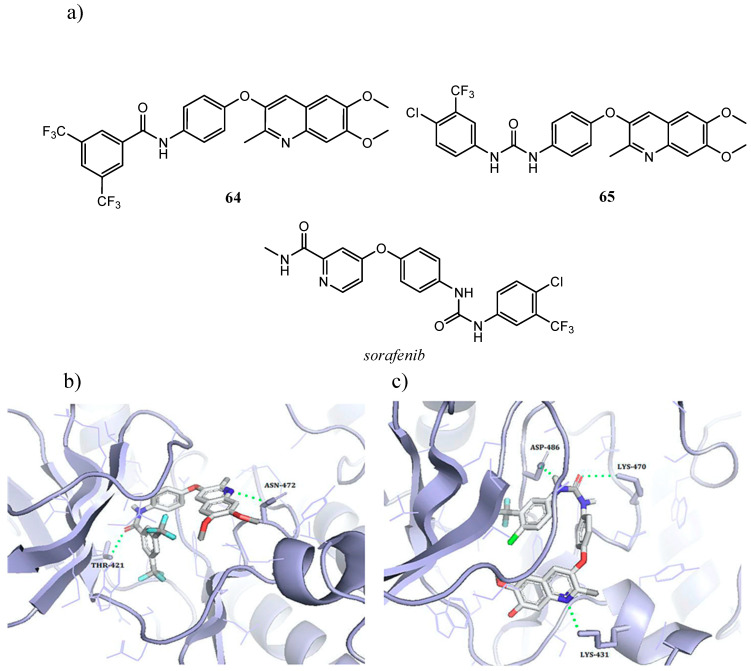
(**a**) Structures of diarylurea derivative *sorafenib* and of quinoline inhibitors **64**,**65** as selective inhibitors of Raf.; (**b**) The best docked pose of **64** within the C-Raf active site (two H-bond stabilize the ligand-protein complex: amidic carbonyl oxygen-Thr^421^, quinolinyl nitrogen-Asn^472^); (**c**) best docked pose for **65** in C-Raf active site (three H-bond stabilize the interaction: urea carbonyl oxygen-catalytic Lys^470^, urea terminal nitrogen-Asp^486^, quinolinyl nitrogen-Lys^431^) [139,140].

El-Gamal et al. synthesized two class of Raf inhibitors possessing quinoline scaffold. In the first one, a diarylamide moiety, through a S or an O atom, was linked at the C-3 of a dimethoxy/dihydroxy-quinoline scaffold. Biological assays realized by National Cancer Institute demonstrated that the most active compounds were dimethoxyquinolines with an oxygen as linker and with electron-withdrawing groups (-Cl or CF_3_) on the terminal ring. One of the most active compounds, **64** (Figure 35a), exhibited GI_50_ values in the micromolar range against the full panel of NCI cancer cell lines and inhibited C-Raf kinase activity by 76.65% at 10 μM [139].

In the second class of quinoline compounds, the same authors substituted the amide linker with urea one to obtain diarylurea derivatives. In NCI five-dose screening protocol, molecule **65**, with the insertion of 4-chloro-3-(trifluoromethyl)phenylurea moiety, was the most promising compound, with a relevant antiproliferative effect compared to the reference diarylurea drug sorafenib (Figure 35a). Inhibition assays of C-Raf kinase evidenced quinoline **65** as more active than the lead compound **64** (for **65** %inhibition at 10 μM = 99.67% and IC_50_ = 0.10 μM). Docking studies of the active site of C-Raf kinase (PDB ID: 3OMV) further explained the high potency of **65** with respect to **64**. Indeed, compound **65**, due to the greater flexibility of the urea linker, formed additional H-bonds with Asp^486^ and Lys^470^ (Figure 35b,c) [140].

El-Damasy et al. designed and synthetized several quinoline analogues of sorafenib, substituting the central phenoxy nucleus with a quinoline one and inserting the two recurrent moieties of the lead compound, the arylurea/arylamide and the N-methyl picolinamide, respectively at the C-2 and C-5 positions of the quinoline ring system.

**Figure 36:**
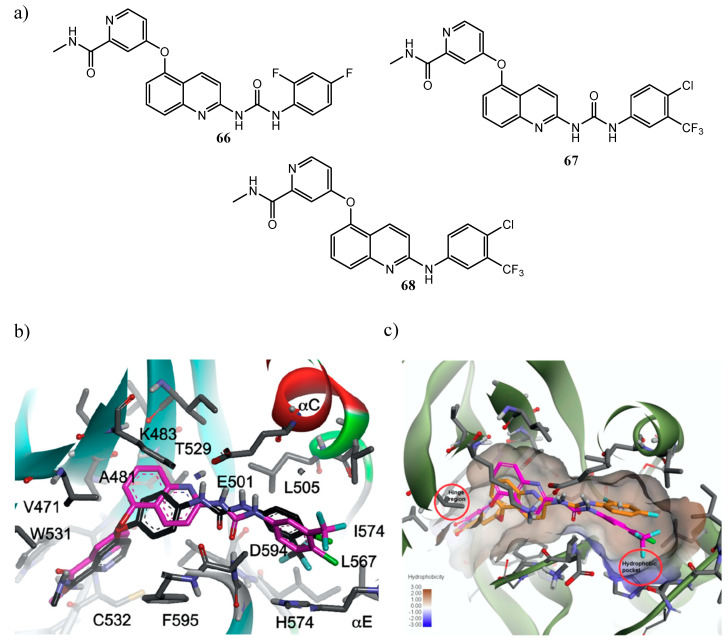
(**a**) Quinoline inhibitors of Raf compared with the reference compound *sorafenib*; (**b**) overlay of the docked pose of compound **67** (magenta) and co-crystalized *sorafenib* (black) in the catalytic kinase domain of BRAF^V600E^ (PDB id: 1UVJ); (**c**) superimposition of the docked pose of compound **67** (magenta) and **66** (orange) in the catalytic kinase domain of BRAF^V600E^ (PDB id: 1UVJ) [141,142].

NCI antiproliferative assays of urea derivatives with fluorinated phenyl ring, as **66** and **67** (Figure 36a), showed GI_50_ values in the low-submicromolar range for the majority of the tested cell lines. Furthermore, compound **67** obtained remarkable results in the kinase inhibition assays, with high selectivity against Raf family and IC_50_ of 316 nM and 61 nM against BRAF^V600E^ and C-Raf, respectively (for the reference sorafenib BRAF^V600E^ IC_50_ = 38 nM and C-Raf IC_50_ = 6 nM); instead of quinoline derivative **66**, that exerted its inhibitory effect only against C-Raf. Docking studies showed a similar binding mode between **67** and sorafenib in the catalytic kinase domain of BRAF^V600E^, indeed both molecules formed interactions through the picolinamide, urea, and trifluoromethylphenyl moieties (Figure 36b); on the other hand, the absence of activity of **66** against BRAF^V600E^ was justified by the unfit orientation of the 2,4-difluorophenyl group, far from the hydrophobic pocket of the allosteric site (Figure 36c) [141]. 

Considering **67** as lead compound, the same authors, by the substitution of the urea linker with an amine one, developed a new series of 2-anilinoquinolines bearing the N-methylpicolinamide group at the C-5 position of the quinoline scaffold. The derivative 4-chloro-3-(trifluoromethyl)aniline **68** (Figure 36a) manifested more remarkable antiproliferative effects than compound **67** and sorafenib, especially against melanoma and breast cancer panels. However, the replacement of the urea linker with an amine in compound **68** has a negative effect on Raf kinase inhibition, that resulted modestly. Docking studies in the catalytic kinase domain of BRAF^V600E^, in fact, underline the capability of quinoline **67** to form additional interactions right through its urea moiety; furthermore, the short amine spacer inhibited the insertion of the 4-chloro-3-(trifluoromethyl)phenyl group within the hydrophobic allosteric site adjacent the ATP binding site, precluding some stabilizing hydrophobic interactions [142].

**Figure 37:**
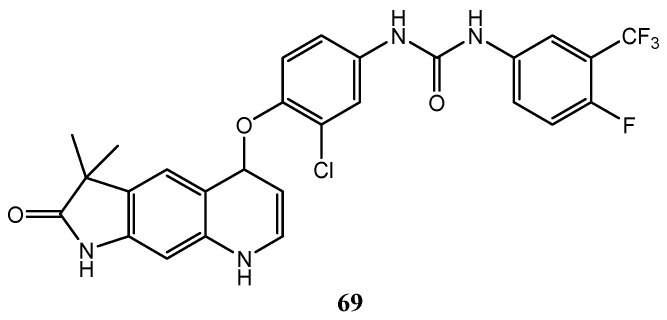
3,3-dimethyl-1*H*-pyrrolo[3,2-*g*]quinolin-2(3*H*)-one derivative as potent C-Raf inhibitor [143].

Li et al. developed quinoline derivative **69** (Figure 37) with urea and a 4-fluoro-3-(trifluoromethyl)phenyl moieties, as sorafenib. It exhibited higher antiproliferative activity than the reference compound, against Hep G2, A549 and KCC-853 cancer cell lines and also a higher inhibitory activity against C-Raf (for **69** C-Raf IC_50_ = 8.7 nM; for sorafenib C-Raf IC_50_ = 28.5 nM) [143].

In the Ras/Raf/MEK signalling cascade, MEK has been also identified as an interesting target for quinoline-based agents able to inhibit its catalytic activity.

**Figure 38:**
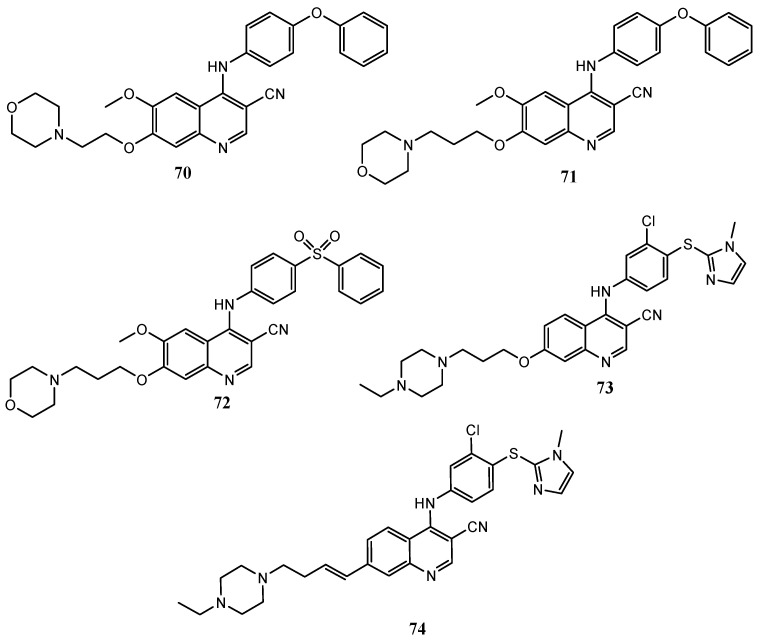
Structures of some MEK inhibitors with 4-anilino-quinoline-3-carbonitrile core [144,145,146,147].

Zhang et al. developed 4-(phenoxyanilino)quinoline **70** (Figure 38), that showed high selectivity against MEK enzyme with an IC_50_ value of 25 nM. SAR studies demonstrated that the cyanoquinoline core is crucial in the inhibition of the enzyme if compared with the less active quinazoline analogues [144]. The same authors also explored the effects of different substitutions on the 4-anilino group in some 4-anilino-3-cyano-6-methoxy-7-(3-morpholino-4-yl-propoxy)quinolines; compounds **71** and **72** (Figure 38), with a phenoxy and phenylsulfonyl substitution respectively, were the most potent (MEK IC_50_ of 7 and 5,7 nM, respectively) [145].

Berger and coworkers discovered some ATP competitive inhibitors of MEK1 kinase with a 6,7-dialkoaxy-3-quinolinecarbonitrile core, as **73** and **74** (Figure 38), in which the 4-anilino moiety is substituted with the 1-methyl-1*H*-imidazol-2-ylsulfonyl one. Compound **73** exhibited a MEK IC_50_ = 3 nM, exceptional in vitro, (IC_50_ = 7 nM), and in vivo activity against LoVo xenografts in nude mice [146]. 

In order to improve the solubility and the oral bioavailability, the same authors further developed molecule **74**, with an alkenyl group at C-7 of the quinoline core; this compound demonstrated good in vitro potency (MEK1 IC_50_ = 12 nM) and high plasma levels after oral dosing in H358 xenograft models [147].

**Figure 39:**
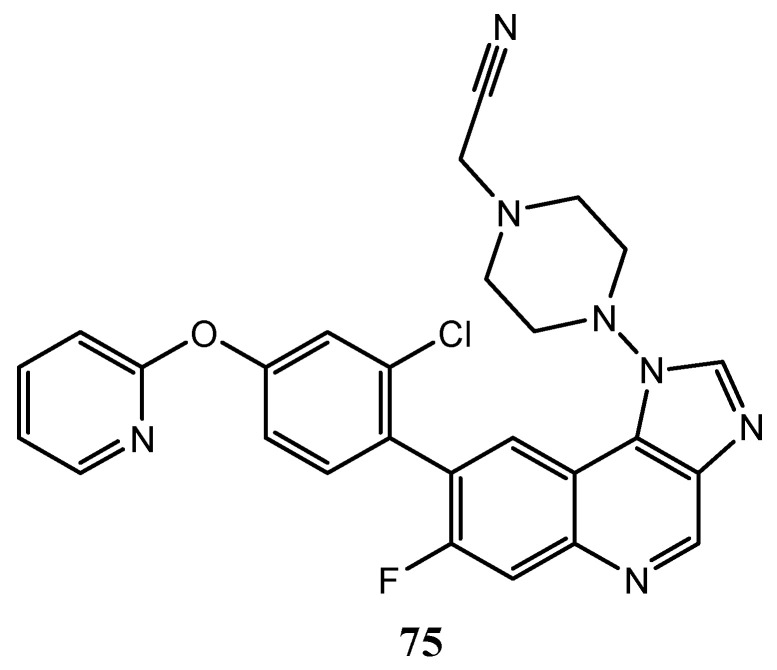
Structure of MEK inhibitor 1*H*-imidazo[4,5-*c*]quinoline **75**.

Polycondensed quinoline systems, as the 1*H*-imidazo[4,5-*c*]quinoline **75** (Figure 39) show interesting results in MEK kinase inhibition assays, with IC_50_ values lower than 100 μM; it was demonstrated that the high selectivity for MEK over other kinases depends on the –F substituent at the C-7 position of the quinoline nucleus [148].

**Figure 40:**
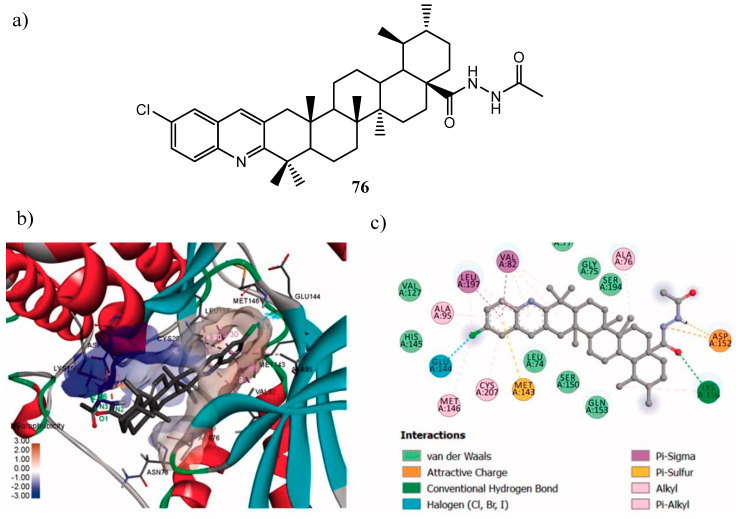
(**a**) structure of the quinoline derivative of ursolic acid **76**; (**b**) binding pose of **76** within MEK1 kinase domain (PDB id: 3EQF); (**c**) ligand interactions of **76** docked into MEK1 active site [149].

An interesting derivative of ursolic acid possessing a condensed chloro-substituted quinoline nucleus and a hydrazide moiety was developed recently by Jin et al. (**76** in Figure 40). Compound **76** showed remarkable results as antiproliferative agent, capability to inhibit MEK1 kinase activity (MEK1 IC_50_ = 64 nM) and activation of Ras/Raf/MEK/ERK pathway. Molecular docking analysis into the MEK1 binding site proved that the ursolic acid skeleton ensured the correct orientation of the hydrazide side chain and of the quinoline ring into the pocket, while the latter were involved in stabilizing interactions with the amino acids [149].

## 3. Conclusions

The quinoline ring system is a scaffold highly frequent in druggable molecules. Currently, a great amount of quinoline derivatives exhibit pharmaceutical activity as antibacterial, antifungal, antimalarial, anthelmintic, local anaesthetic, antipsychotic, and anticancer drugs.

In the anticancer field, the quinoline nucleus has been observed as crucial molecular moiety recurrent in several inhibitors of kinases, for the treatments of a wide range of tumours in the targeted therapeutic approach.

In particular, this review summarizes the biological data of several quinoline compounds, which are active on c-Met, VEGF, and EGF receptors, and on the related proteins involved in the intracellular signalling cascades. Overexpressed in tumour cells, these three protein kinases receptors trigger carcinogenic pathways closely connected with each other, regulating the survival processes in the cell, such as proliferation, apoptosis, differentiation, and angiogenesis.

The druggable attitude of the quinoline scaffold probably lies on its proved biocompatibility, versatility, and chemical accessibility, from which new derivatives can be easily designed and synthesized.

The quinoline nucleus is an electron-deficient ring system with tertiary base properties. The presence of nitrogen withdraws electrons by resonance, interfering with the equal distribution of the π-electron density and suggesting a chemical behaviour similar to the pyridine. 

In view of an SAR study, the analysis of the interactions of the quinoline molecules in the protein binding sites, under investigation, highlights recurrent hydrogen bonds with the nitrogen of the quinoline ring and π-π stacking complexes with complementary amino acid residues. The presence of flexible moiety, especially at C-4, C-6, and C-7 positions, frequently consolidates the force of the ligand–protein complexes. 

All the biological data of the quinoline compounds, analysed in the present review, are reported in detail, confirming the antiproliferative activity and the pivotal importance of this ring system in the efficacy of several approved drugs. In drug discovery, the information recovered in this manuscript could help in the design and rationale optimization studies for the development of new quinoline multitargets molecules.

## Figures and Tables

**Table 1:** Examples of some interesting quinoline based c-Met inhibitors and their corresponding biological activities.

Quinoline Compound	Linker	IC_50_ (μM) Sensitive Cell Lines	IC_50_ on c-Met (nM)	Ref.
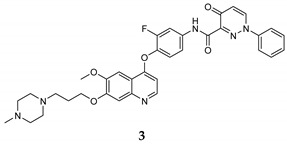	pyridazinone-3- carboxamide	A549 (IC_50_ = **0.003** **±** **0.001**) HepG2 (IC_50_ = **0.49** **±** **0.003**) MCF-7 (IC_50_ = **0.006** **±** **0.001**)	**0.6**	[35]
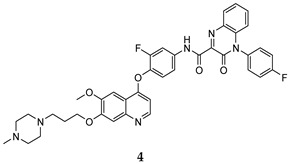	3-oxo-3,4- dihydroquinoxaline	HT-29 (IC_50_ = **0.06 ± 0.001**) A549 (IC_50_ = 0.050 ± 0.006) H460 (IC_50_ = **0.18 ± 0.01**) MKN-45 (IC_50_ = **0.023 ± 0.005**) U87 MG (IC_50_ = **0.66 ± 0.09**)	**0.9**	[36]
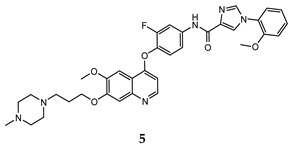	1H-imidazole-4- carboxamide	HT-29 (IC_50_ = **0.08 ± 0.02**) MKN-45 (IC_50_ = **0.22 ± 0.03**) A549 (IC_50_ = **0.07 ± 0.01**)	**1.1 ± 0.21**	[37]
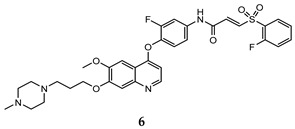	(E)-3-hydrosulfonyl acrylamide	HT-29 (IC_50_ = **0.15 ± 0.003**) MKN-45 (IC_50_ = **0.28 ± 0.003**) A549 (IC_50_ = **0.15 ± 0.05**)	17 ± 0.20	[37]
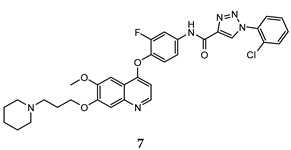	1,2,3-triazole-4- carboxamide	HT-29 (IC_50_ = **0.10 ± 0.003**) H460 (IC_50_ = **0.18 ± 0.03**) A549 (IC_50_ = **0.07 ± 0.01**) MKN-45 (IC_50_ = **0.03 ± 0.01**)	2.27	[38]
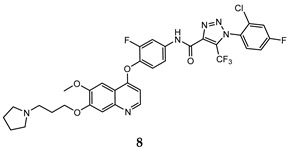	1,2,3-triazole-4- carboxamide	A549 (IC_50_ = **0.08 ± 0.004**) H460 (IC_50_ = **0.15 ± 0.03**) HT-29 (IC_50_ = **0.12 ± 0.02**) MKN-45 (IC_50_ = **0.021 ± 0.003**) U87MG (IC_50_ = **0.85 ± 0.12**)	**1.04 ± 0.09**	[39]
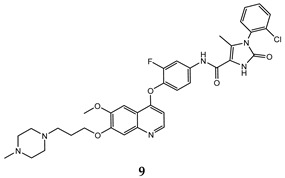	2-imidazolone-4- carboxamide	A549 (IC_50_ = 0.25 ± 0.02) H460 (IC_50_ = **0.10 ± 0.008**) HT-29 (IC_50_ = 0.086 ± 0.005) MKN-45 (IC_50_ = **0.014 ± 0.004**)	**1.42 ± 0.14**	[40]
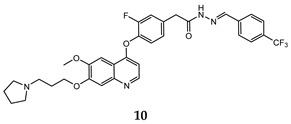	acylhydrazone	HT-29 (IC_50_ = **0.15 ± 0.03**) H460 (IC_50_ = **0.031 ± 0.008**) MKN-45 (IC_50_ = 0.37 ± 0.08) A549 (IC_50_ = **0.080 ± 0.01**) U87MG (IC_50_ = **0.29 ± 0.08**)	1.86	[41]
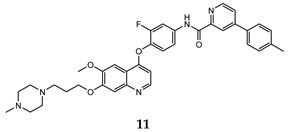	pyridine -2-carboxyamide	HT-29 (IC_50_ = **0.026 ± 0.003**) H460 (IC_50_ = **0.037 ± 0.01**) MKN-45 (IC_50_ = 0.073 ± 0.01) A549 (IC_50_ = **0.10 ± 0.02**) U87MG (IC_50_ = **0.81 ± 0.19**)	1.39	[42]
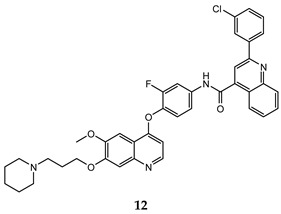	2-phenylquinoline-4- carboxamide	H460 (IC_50_ = **0.011 ± 0.002**) HT-29 (IC_50_ = **0.062 ± 0.01**) MKN-45 (IC_50_ = **0.011 ± 0.003**) U87MG (IC_50_ = **0.15 ± 0.03**) SMMC-7721 (IC_50_ = **0.03 ± 0.004**)	1.32	[33]
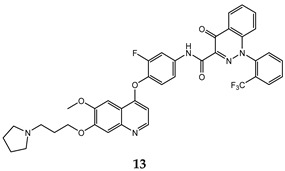	4-oxo-1,4-dihydrocinnoline-3-carboxamide	A549 (IC_50_ = **0.035 ± 0.0042**) H460(IC_50_ = **0.055 ± 0.010**) HT29 (IC_50_ = **0.11 ± 0.016**) MKN-45 (IC_50_ = **0.022 ± 0.0035**) U87MG (IC_50_ = **0.35 ± 0.060**) SMMC-7721 (IC_50_ = **0.25 ± 0.072**)	**0.59 ± 0.07**	[43]
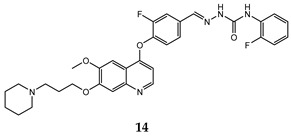	acyclic semicarbazone	HT-29 (IC_50_ = **0.059 ± 0.0039**) MKN-45 (IC_50_ = 0.016 ± 0.0012) A549 (IC_50_ = **0.0090 ± 0.0012**) MDA-MB-231 (IC_50_ = 0.77 ± 0.064)	4.3	[44]
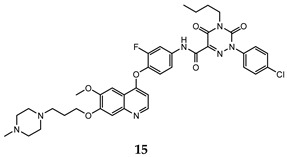	l,2,4-triazine-3,5-dione	H460 (IC_50_ = 0.14) HT-29 (IC_50_ = 0.21) MKN-45 (IC_50_ = 0.066) SMMC-7721 (IC_50_ = 0.73)	6.3	[47]
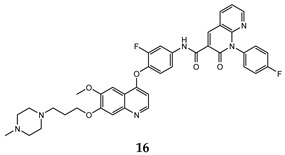	1,8-naphthyridin-2-one	HepG2 (IC_50_ = **0.23 ± 0.01**) MCF-7 (IC_50_ = **0.42 ± 0.06**) A549 (IC_50_ = **0.21 ± 0.02**)	2.36	[48]
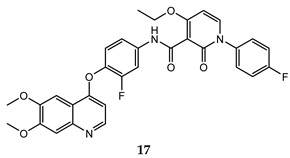	2-oxo-1,2-dihydropiridine-3-carboxamide	MKN45 (IC_50_ = **0.039 ± 0.009**) BaF3/TRP-Met ((IC_50_ = **0.019 ± 0.0088**)	0.9 ± 0.1	[49]
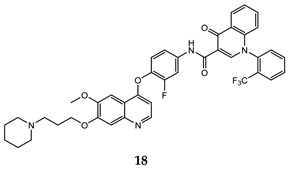	4-oxo-1,4-dihydroquinoline-3-carboxamide	H460 (IC_50_ = **0.075**) HT-29 (IC_50_ = **0.051**) MKN-45 (IC_50_ = **0.010**) U87MG (IC_50_ = **0.53**) SMMC-7721 (IC_50_ = **0.20**)	1.35	[50]
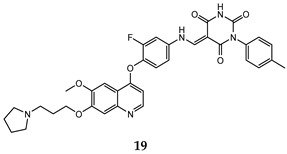	5-(aminomethylene) pyrimidine-2,4,6-trione moiety	HT-29 (IC_50_ = **0.13 ± 0.060**) H460 (IC_50_ = **0.051 ± 0.0080**) MKN-45 (IC_50_ = 0.057 ± 0.0040) A549 (IC_50_ = **0.072 ± 0.0060**) U87MG (IC_50_ = **0.64 ± 0.045**)	**1.15**	[51]
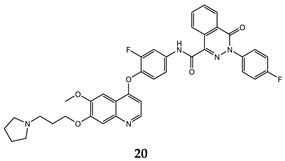	4-oxo-3,4-dihydrophthalazine-1-carboxamide	H460 (IC_50_ = **0.055 ± 0.010**) MKN-45 (IC_50_ = 0.071 ± 0.011) HT-29 (IC_50_ = **0.13 ± 0.016**) MDA-MB-231 (IC_50_ = **0.43 ± 0.050**)	1.63	[52]
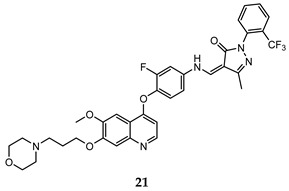	pyrazolone	HT-29 (IC_50_ = **0.14 ± 0.02)** H460 (IC_50_ = **0.18 ± 0.03**) A549 (IC_50_ = **0.09 ± 0.02**) MKN-45 (IC_50_ = **0.03 ± 0.001**) U87MG (IC_50_ = **1.06 ± 0.05**)	2.20	[53]
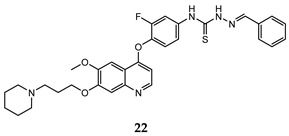	(thio)semicarbazones	A549 (IC_50_ = 0.33 ± 0.02) HT-29 (IC_50_ = **0.21 ± 0.02**) MKN-45 (IC_50_ = 0.71 ± 0.08) MDA-MB-231 (IC_50_ = 1.2 ± 0.17)	8.92	[54]

**Table 2:** *Omipalisib* and analogues **31**–**34** selective active on PI3Kα and mTOR.

Quinoline Compound	Inhibition Data (IC_50_ or GI_50_) In Antiproliferative Assays	Enzymatic Inhibition Data (IC_50_ or K_i_)	Ref.
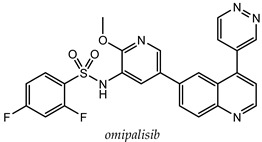	T47D (IC_50_ = 3 nM) BT474 (IC_50_ = 2.4 nM)	PI3Kα IC_50_ = 0.04 nM; mTORC1 K_i_ = 0.18 nM; mTORC2 K_i_ = 0.3 nM	[67]
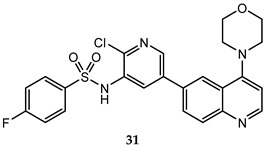	U-87 MG (IC_50_ = 16 ± 5.8 nM)	PI3Kα (IC_50_ = 4.6 ± 3 nM; K_i_ = 0.6 ± 0.5 nM); mTOR IC_50_ = 3.9 ± 1 nM	[69]
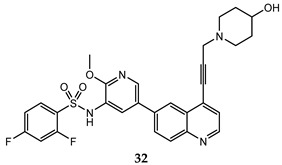	PC-3 (IC_50_ = 0.37 μM); HCT-116 (IC_50_ = 2.47 μM)	PI3Kα IC_50_ = 1.63 nM mTOR IC_50_ = 3.6 nM	[70]
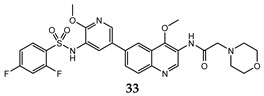	PC-3 (IC_50_ = 0.08 μM)); HCT-116 (IC_50_ = 0.47 μM)	PI3Kα IC_50_ = 1.7 nM mTOR IC_50_ = 10.0 nM	[71]
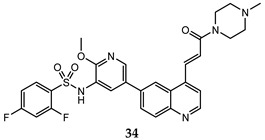	PC-3 (GI_50_ = 0.40 μM); HCT-116 (GI_50_ = 0.47 μM)	PI3Kα IC_50_ = 0.50 nM mTOR IC_50_ = 1.3 nM	[72]

**Table 3:** Quinoline derivatives **35–37** with interesting activity on both mTORC1 and mTORC2.

Compound	Antiproliferative Assays (IC_50_, μM)	mTOR Inhibition Data (IC_50_, nM)	Ref.
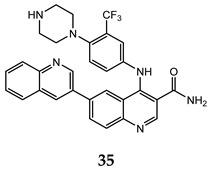	HCT-116 (IC_50_ = 0.46 μM) PC-3 (IC_50_ = 0.61 μM) MCF-7 (IC_50_ = 0.24 μM)	14	[74]
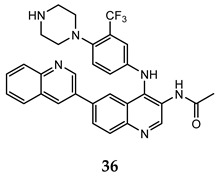	HCT-116 (IC_50_ = 0.36 μM) PC-3 (IC_50_ = 0.50 μM) MCF-7 (IC_50_ = 0.11 μM)	22	[75]
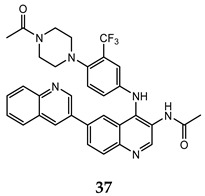	HCT-116 (IC_50_ = 0.11 μM) PC-3 (IC_50_ = 0.17 μM) MCF-7 (IC_50_ = 0.05 μM)	30	[75]
